# Extra-Framework Anion Species in Scapolites Exhibiting Natural and Thermally Induced Photoluminescence: Insights from Single-Crystal XRD

**DOI:** 10.3390/ma19214623

**Published:** 2026-10-30

**Authors:** Vladislav Kostov-Kytin, Rositsa Kukeva, Petia Petrova, Rositsa Titorenkova, Nadia Petrova, Boris Shivachev, Rositsa Nikolova

**Affiliations:** 1 Institute of Mineralogy and Crystallography “Acad. Ivan Kostov”, Bulgarian Academy of Sciences, Blvd. Acad. G. Bonchev Str., Bld. 107, 1113 Sofia, Bulgaria; rositsatitorenkova@imc.bas.bg (R.T.); nadia5@mail.bg (N.P.); blshivachev@gmail.com (B.S.); 2 National Centre of Excellence “Mechatronics and Clean Technologies”, 8 Blvd. Kliment Ohridski, P.C. 1756 Sofia, Bulgaria; 3 Institute of General and Inorganic Chemistry, Bulgarian Academy of Sciences, Blvd. Acad. G. Bonchev Str., Bld. 11, 1113 Sofia, Bulgaria; kukeva@svr.igic.bas.bg; 4 Institute of Optical Materials and Technologies “Acad. Jordan Malinowski”, Bulgarian Academy of Sciences, Blvd. Acad. G. Bonchev Str., Bld. 109, 1113 Sofia, Bulgaria; petia@iomt.bas.bg; 5 PERIMED-2, BG16RFPR002-1.014-0007, Central District, Vasil Aprilov Blvd. 15A, 4002 Plovdiv, Bulgaria

**Keywords:** scapolite, photoluminescence, thermally induced luminescence, sulfur-related defects

## Abstract

**Highlights:**

- A new interpretation of the electron density distribution in the scapolite anion cage.
- A methodology to determine the quantities of SO_4_^2−^ and CO_3_^2−^ in the scapolite cage.
- Comprehensive data on species diversity and transformations in the scapolite cage.
- The conditions necessary for the formation of S_2_^•–^ radicals in fluorescent scapolites.

**Abstract:**

This study presents results from comprehensive investigations of scapolite samples exhibiting natural or thermally induced photoluminescence. A range of analytical techniques, including single-crystal X-ray diffraction, Fourier-transform infrared spectroscopy, Raman spectroscopy, photoluminescence spectroscopy and electron paramagnetic resonance spectroscopy, were employed to characterize sulfur-related defect centers within the scapolite anionic cages. Differential scanning calorimetry combined with thermogravimetry and mass spectrometry was used to analyze the stages of degassing and quantify the volatile components. A new interpretation of the electron density distribution within the scapolite anionic cage is proposed. This enables the localization of possible precursors of S_2_^•−^ radicals, the latter acting as luminescent centers. Key parameters, including atomic positions, occupancies, and bond lengths, were determined for the newly observed moieties. Furthermore, a novel methodological approach was developed to quantify SO_4_^2−^ and CO_3_^2−^ ratios in the structure based on the occupancy of their oxygen sites. Finally, the conditions necessary for the formation of persistent S_2_^•−^ radicals in fluorescent scapolites are discussed, with an emphasis on the required cationic environment and charge-transfer conditions.

## 1. Introduction

Minerals of the scapolite group are common rock-forming aluminosilicates that occur in a wide variety of metamorphic and altered igneous rocks. Their wide distribution in metamorphic terrains, as well as their high stability over a wide range of pressures and temperatures, makes scapolites potential geothermometers and geobarometers, providing the opportunity to obtain information on the redox potential Eh (from Fe^n+^ valence) and pH (from anion speciation) of their formation [1]. Scapolites store volatiles in the lower crust and upper mantle and are indicators of the activities of volatile components, indicating that they may play a natural role in the capture and storage of greenhouse gases [2]. In the second half of the 20th century, it was widely accepted that natural scapolites form a solid-solution series between the two theoretical end-members: Na_4_Al_3_Si_9_O_24_Cl (marialite) and Ca_4_A1_6_Si_6_O_24_CO_3_ (meionite). In 1998, a third end-member of this group of minerals was approved under the name silvialite, Ca_4_A1_6_Si_6_O_24_SO_4_ [3].

In recent years, in connection with the preparation of new functional materials, researchers have paid increasing attention to materials that exhibit photochromism and/or photoluminescence. Such phenomena are observed in some of the members of the scapolite group as well as in some of the crystallochemically related feldspathoids of the cancrinite and sodalite groups [4,5,6,7,8,9]. The interpretation that yellow–orange emission in natural and synthetic scapolite involves (di)sulfide radicals incorporated into the crystal structure and forming photoluminescence centers is increasingly supported by experimental and theoretical evidence [5,6,10,11,12,13].

Previous studies on scapolite samples from Bulgarian localities as well as from some world-famous deposits of the mineral such as Slyudyanka (Ural, Russia) and Madagascar (private collection) address issues related to the choice of space group for these compounds [14]. The results of preliminary mineralogical characterization of scapolite exhibiting characteristic yellow photoluminescence under long-wave ultraviolet light (LW UL) from the Central Rhodopes, Bulgaria, were recently reported [15]. These findings initiated further single-crystal X-ray diffraction (SCXRD) experiments, aiming at elucidating the structural origin of the luminescence of scapolites. Special attention was paid to the extra-framework species and, in particular, to the atomic configurations in the anionic part of these compounds, which were supposed to contain the sulfur species responsible for the observed phenomenon. New interpretations of the observed electron densities have been made, which have led to revisions of our previous approach to specifying the anionic species in the scapolite crystal structure.

The present work reports the results of comprehensive studies of natural scapolite samples from Bulgarian deposits that exhibit natural or thermally induced photoluminescence. The SCXRD studies play a key role and aim to provide possible evidence for and information on the presence, orientation, and geometry of sulfur dimers in scapolite structures. A combination of spectroscopic methods was further employed to search for sulfur-related defect centers located in the extra-framework cage: infrared (IR) absorption, Raman scattering, photoluminescence (PL) emission, and electron paramagnetic resonance (EPR). Differential Scanning Calorimetry–Thermogravimetry (DSC-TG), accompanied by mass spectroscopy (MS) data, provided information about the type, stages of separation, and quantitative relationships of the volatile components in the studied samples.

## 2. Previous Works

### 2.1. General Notes on the Crystal Chemistry of Scapolites

The general formula of this mineral group is M_4_[T_12_O_24_]A. M designates interstitial framework cation (IFC)-type cations, which are predominantly Ca^2+^ and Na^+^ but may also consist of K^+^, Mg^2+^, Sr^2+^, Ba^2+^, and traces of other elements such as Fe^2+^, Mn^2+^, etc. T stands for tetrahedrally coordinated cations, which are mainly Si^4+^ and Al^3+^, and less commonly Fe^3+^ [16]. Together with the framework oxygen atoms, this group of cations is further designated as framework atoms (FRAs). A represents an impressive diversity of negative ions, anionic groups, molecules and radicals. These are most often Cl^−^, (CO_3_)^2−^ and (SO_4_)^2−^; hydrogen can also be present in various forms, such as HCO_3_^−^, HSO_4_^−^, OH^−^, H_2_O, etc. [17]. The presence of S_2_^•−^ centers, which are responsible for the yellow fluorescence of scapolite, has been reported [18]. These species are further designated as interstitial framework anions (IFAs). There are three types of heterovalent isomorphism for scapolite group members, generally represented as follows: Si⇿Al (FRAs); Ca⇿Na (IFCs); and CO_3_⇿SO_4_⇿Cl (IFAs). The relations between the three schemes are not always explicit and unambiguous, but essentially, their joint manifestation leads to electroneutrality. The meionite component (Me, %) of scapolite in the marialite–meionite series has been defined as Me% = 100 × Ca/(Na + Ca) [19], which is still in active use by researchers.

**Figure 1:**
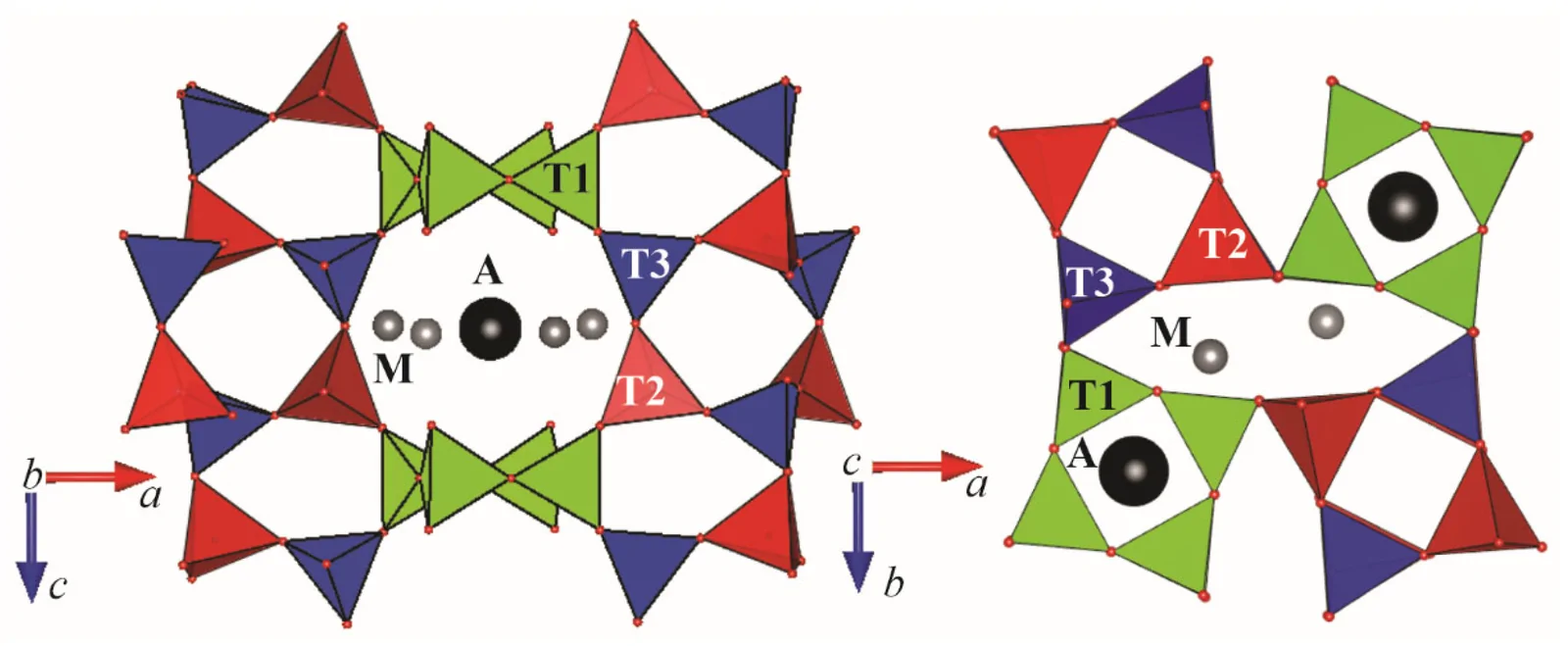
View of scapolite anion cage containing the interstitial framework cations (M) and anions (A) [14].

In a simplified version, the crystal structure of scapolites can be represented as composed of a [T_12_O_24_] framework, containing eight- and four-membered rings of tetrahedra. IFCs are located in oval-shaped channels, formed by the eight-membered rings; IFAs inhibit channels formed by the four-membered rings. Together, IFCs and IFAs occupy space in and around the center of the scapolite anion cage (SAC) [20,21] (Figure 1).

Typically, the scapolite crystal structure is described in tetragonal *I*4/*m* or *P*4_2_/*n* space groups. Recent studies, however, give reason to assume that a more correct choice of space group for describing the structures in the entire range of scapolites is *P*4_2_/*n* [14]. 

### 2.2. Approaches to Solving the Crystal Structures of Scapolites from X-Ray Diffraction Data

Pauling proposed the first model for the crystal structure of scapolite [22]. The research groups that have devoted efforts to solving the crystal structures of various members of this group of minerals are listed by [16]. A brief overview of the approaches to addressing the challenges of the scapolite structure related to the three groups of atoms (FRAs, IFCs, and IFAs) is given below:

(i) Framework atoms (FRAs)—determination of the Si/Al ratio: Since the scattering factors of silicon (Z = 14) and aluminum (Z = 13) are similar, it is well known that the XRD method cannot be used directly to refine the T-site occupancies. Instead, the difference in ionic radius between tetrahedrally coordinated aluminum and silicon is used, as this directly affects the T–O bond lengths, and, therefore, the Si and Al occupancies at each tetrahedral site can be evaluated and assigned [14]. Moreover, the FRAs’ crystallographic sites of scapolite are assumed to be fully occupied (100%).
(ii) Interstitial framework cations (IFCs)—population and occupancy: Various factors influencing IFCs’ characteristics have been reported [14,16,23,24,25]. The usual practice is to fix the occupancy of these cation sites according to their chemical composition, as determined by independent analytical methods. The presence of vacancies cannot be ruled out [16]. Sometimes, when processing chemical analyses, certain negligible amounts of detected di- and monovalent extra-framework cations together with possible vacancies have been treated as a monovalent fictive cation M+ in the structural refinements [14].
(iii) Interstitial framework anions (IFAs)—atomic model derivation: The contents of C, Cl, and S obtained by chemical analyses are presented as CO_3_^2−^, Cl^−^, and SO_4_^2−^. Because electron microprobe (EMP) carbon measurements are prone to contamination, carbon content is typically calculated using charge balance or simplified empirical formulas. SCXRD studies indicate that anionic groups are located in the SAC [14,16,20,21,25,26,27,28,29].

**Figure 2:**
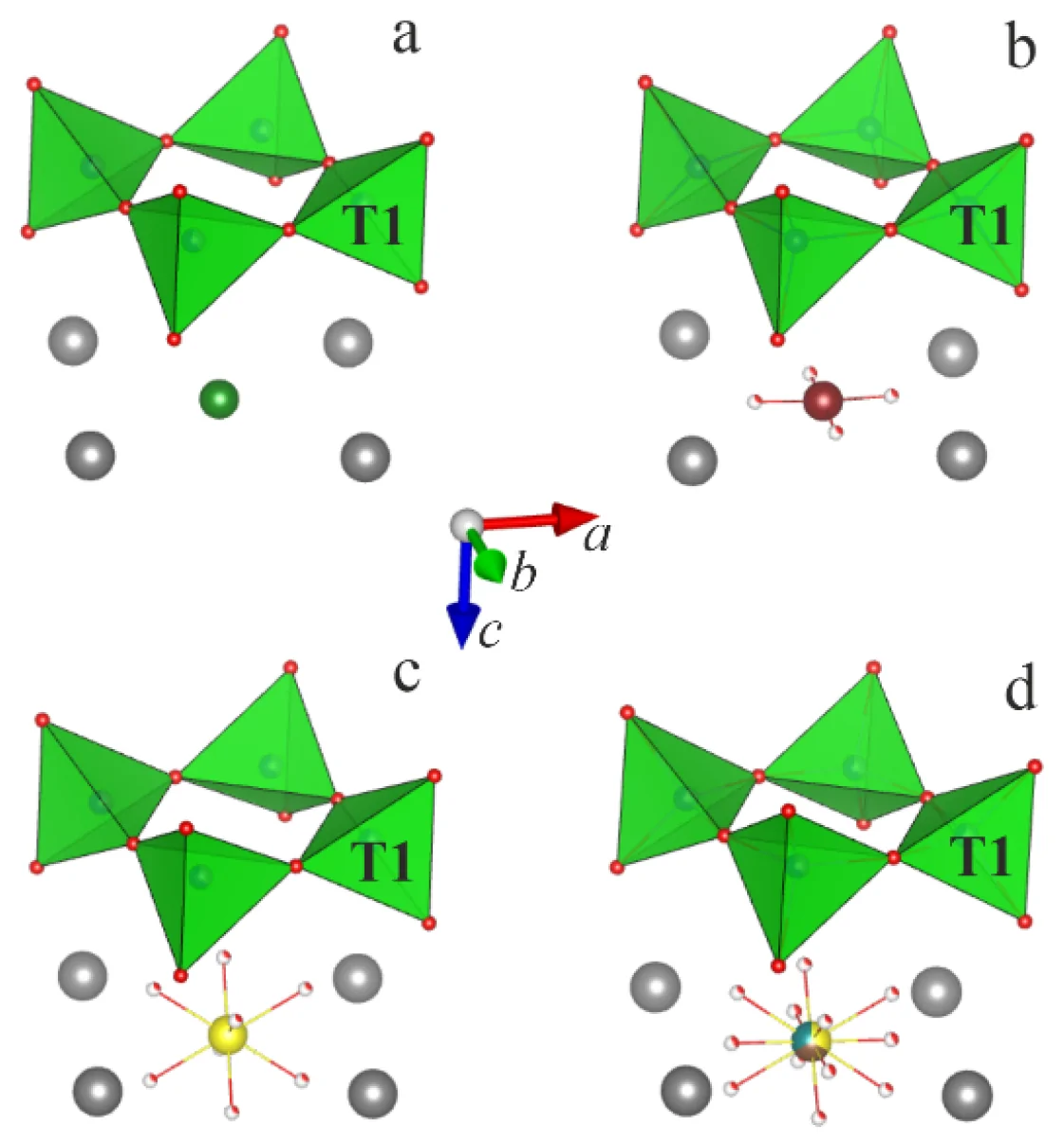
Atomic configurations characteristic of the A-position and its vicinity in the structure of scapolite: (**a**)—Cl^−^ (green); (**b**)—CO_3_^2−^ (C—brown); (**c**)—SO_4_^2−^ (S—yellow); and (**d**)—combined view of all species. Other designations and crystallographic orientations are similar to those in Figure 1.

In predominantly chlorine-containing samples, a single Wyckoff position (2a or 2b) is identified in the electron density distribution map (Figure 2a). In carbonate- or sulfate-bearing samples, additional electron density appears around this axis, lying on or outside the plane perpendicular to the four-fold axis and at distances corresponding to C–O or S–O bonds in carbonate or sulfate groups. This distribution of electron density in the SAC is interpreted as follows: In the position lying on the four-membered axis, chlorine, carbon or sulfur is positioned, and in the positions located around the four-membered axis, the carbonate and sulfate oxygen atoms are placed (Figure 2b,c). This interpretation cannot account for the trigonal symmetry of the carbonate group. A model was proposed in which the carbon atom is slightly offset by about 0.3 Å from the four-fold axis, and in addition, the density located at a distance of 1.35 Å from the carbon is distributed over three symmetry-independent positions so as to obtain four carbonate groups around the four-fold axis [21,26]. This structural model requires fixing the geometric parameters of the carbonate group during refinement. It is important to note, however, that in the majority of published crystal structures, no splitting of either the central or oxygen positions is reported, e.g., in the structure of natural silvialite [3].

### 2.3. Cause for the Yellow–Orange Photoluminescence of Scapolites

The coloration and luminescent properties of scapolites have long attracted the attention of both jewelers and researchers seeking to unravel the mechanisms that cause them. In one of the early reports on this subject, Shaw suggested that the strong fluorescence of the yellow scapolite from Grenville, Quebec, was due to the presence of small amounts of uranium, which is the activating agent [19]. Over the years, it has been established that, even with low contents, other extra-framework cations (Fe^3+^, Mn^2+^, and Ce^3+^) can also cause luminescence of various colors [8,9]. Recently, more researchers have come together around the opinion that the characteristic yellow–orange photoluminescence of scapolites is associated with S_2_^•−^ centers [10,18,30]. In 2008, Sidike et al. reported that the relative efficiency of yellow photoluminescence from a natural scapolite sample investigated by them was increased up to 117 times by heat treatment at 1000 °C for 2 h in air. The enhancement of yellow photoluminescence was ascribed to the alteration of SO_3_^2−^ and SO_4_^2−^ to S_2_^•−^ [18].

The important characteristics of such sulfur dimers (either S_2_^2−^ or S_2_^•−^) having direct relevance to the present studies are (i) position, (ii) quantity, (iii) orientation, and (iv) S-S bond length in scapolite structures.

(i) Typically, SACs are accepted as the location of S_2_^•−^ centers. The latter are surrounded by IFCs in a square configuration (IFC square).

(ii) So far, no specific data have been reported on the concentrations of sulfur dimers in the structures of natural scapolites exhibiting yellow photoluminescence. The possible reasons for this are low amounts of the registered element; the possibility of the co-presence of several sulfur species; and previously applied methodologies for determining IFAs by SCXRD. One should always take into account general considerations for photoluminescence activator concentrations, the increase in which can lead to enhancement of this phenomenon, as has already been observed [18]. It is, however, also possible to achieve “concentration quenching” and inhibition of photoluminescence.

(iii) Two very different views have been reported regarding the structural orientation of the sulfur dimer in scapolites. One of them presented the S_2_^•−^ centers in an anion cage as S-S pairs that lie along the diagonal of the IFC square planes [18,31]. The other one states that this is incorrect, and the sulfur dimer is located perpendicular to the IFC square [10].

(iv) The S-S bond lengths for S_2_^•−^ species in natural and synthetic scapolites were calculated using the vibronic structure of the emission spectra, applying Steudel’s formula [10,13]. The obtained values fall within the range of 1.99–2.04 Å (mean value: 2.02 Å). Data for interatomic distances in the sulfur dimer in other inorganic compounds can be obtained by calculations or direct measurements. For example, values in the range ~2.03 to ~2.36 Å, with the caveat that they depend strongly on the nature of the counter-cation and on the physical properties of the crystal, were reported [32]. Distances varying from 2.1 to 2.4 Å were measured for monoclinic lazurite containing several sulfur species, such as SO_4_^2−^, SO_2_, and S_2_ groups [33]. A S−S (S_2_^2−^) bond length of 2.13 Å was reported for S-doped sodalites [11]. The authors, however, noted that under UV irradiation, charge transfer occurred, and that the optimized geometry revealed a shorter S−S distance (2.00 Å).

## 3. Materials and Methods

### 3.1. Materials and Heat Treatment Procedure

In this work, natural scapolite samples from two localities situated in the Rila-Rhodope massif in Bulgaria were investigated. These were the Urdini Lakes (ULs) in the Northwestern Rila Mtn. (Me ~ 70%) [34] and Mishevsko (Msh), Central Rhodopes (Me > 60%) [15]. While the UL samples were massive and relatively homogeneous in composition (with plagioclase and zeolite below 10%), the Msh scapolites were highly fractured and intimately intergrown with other phases such as diopside, plagioclase and calcite (total of up to 50%).

The untreated Msh samples exhibited characteristic yellow photoluminescence under LW UV excitation, whereas the untreated samples from the ULs showed no detectable photoluminescence under the same conditions. Thermal treatment of the samples from both localities was undertaken in accordance with Sidike’s work in an attempt to verify the sulfur-species alteration proposed by the authors [18]. Therefore, the heat treatment was performed in Carbolite Gero CWF 1300 laboratory (Hope, Derbyshire, United Kingdom) chamber furnaces at 1000 °C for 1 h in air at a heating rate of 10 °C/min and sample weights of 1–5 g. After heating, the samples from the Urdini Lakes developed yellow photoluminescence under UV excitation, with luminescent areas constituting less than 20%. The induced luminescent state was persistent and, in the present case, remained observable for more than five months after thermal treatment (see Supplementary Materials Figure S1).

### 3.2. Single-Crystal X-Ray Diffraction (SCXRD)

Single-crystal analyses were performed on at least 10 heated and unheated samples from each locality. Crystals with dimensions of approximately 0.05 × 0.05 × 0.05 mm were mounted on a glass capillary and used for single-crystal diffraction analysis. All the measured Msh crystals and only part of the heated UL samples exhibited photoluminescence. Data collection experiments were performed with a Bruker D8 Venture diffractometer (MoKα(0.7107 Å)), both under room-temperature (RT; 273 K) and low-temperature (LT; 150 K) conditions. The determination of unit cell parameters, data integration, scaling and absorption corrections were performed using the APEX6 v2024.9-1 software package. All the crystal structures were solved by direct methods and refined by full-matrix least-squares methods on F^2^ using the SHELXT 2018/2 software program [35].

In the present work, particular attention was focused on the most reliable determination of the positions and quantitative ratios of the anionic components. Our approach was based on consistency with the experimental electron density distribution, while minimizing the use of constraints in the structural model. The following considerations were taken into account when positioning the anionic components: All atoms building up the structure of scapolite, except for the oxygen atoms in the carbonate group, are subject to tetragonal symmetry. The latter, however, contribute negligibly to the value of the structural factors, because they are a very small part of the stoichiometric formula of scapolite, and, even in the ideal case, Ca_4_Al_6_Si_6_O_24_CO_3_, represent 5% by weight. Accordingly, when calculating the electron density distribution map based on the experimental data, the information about the trigonal symmetry of the carbonate oxygen atoms remains “masked” by the impact of the rest of the atoms. Chlorine, carbonate carbon, and sulfate sulfur atoms occupy one and the same crystallographic site: the A-position.

The steps we followed to determine and refine the crystal structure are listed below. Explanations are also provided. At each step, the parameters from the previous one were refined.

**Figure 3:**
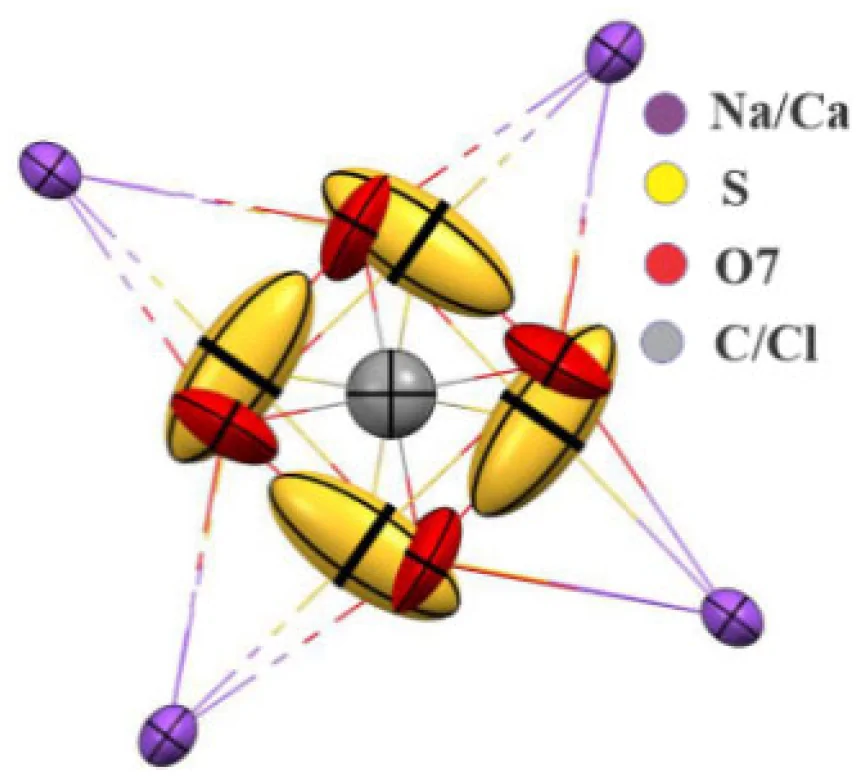
Displacement ellipsoids of IFAs and IFCs for the untreated Msh sample.

(i) From the initial electron density distribution map, the positions of FRAs and IFCs and the position of the central atom of the anionic groups, as well as those of the oxygen atoms of the IFA species (e.g., CO_3_^2−^ and SO_4_^2−^), were assigned. In this step of the structural model, only Si, Ca, and Cl for the FRA cations, IFCs, and the central atom of the anionic groups, respectively, were used. The further procedure included refinement of all atomic positions and the occupancies of IFC and IFA sites, assuming unity for all FRA positions. The refined occupancies invariably fell below 100%.

(ii) The Al/Si ratio in the three symmetrically independent tetrahedra (solely using *P*4_2_/*n*) was fixed in compliance with the measured averaged T–O distances. Upon acceptance of 100% occupancy for the IFC positions, the Ca/Na ratio was refined.

(iii) Chlorine, carbon, and sulfur were placed in the A-position. The initial amounts of carbon and sulfur were determined in accordance with the occupancies of the surrounding oxygen atoms obtained in the previous step. In a couple of cycles, anisotropic displacement parameters of FRAs and IFCs, as well as the amounts of Cl, CO_3_^2^, and SO_4_^2^, were refined. As a result, it was found that the A-position in all of the studied samples was filled to about 80–90%, and residual electron density (1–1.5 e^−^ Å^−3^) was observed at a distance of 1.06–1.15 Å from the central A-position. Such positive peak values are borderline. This suggests either their disregard as a possible atomic position or their assignment to a specific species.

(iv) In accordance with the results of other analytical methods, our choice was to place sulfur in this position. The tetragonal symmetry of the studied object predetermined four such symmetry-equivalent sites confined to the ab-plane. For the selected samples, the distance between opposite positions in this configuration varied from 2.09(5) to 2.28(6) Å. This geometry is best described by the presence of a sulfur dimer (S_2_^2–^ or S_2_^•−^). The final refinement included isotropic displacement parameters and the occupancy of this position, which ranged from 0.053(13) to 0.091(12). We only refined the isotropic parameters for this position because the anisotropic parameters showed significantly higher values than those of the other atoms in the structure. However, it is worth noting that in all studied samples, the thermal ellipsoids at this position were strongly elongated along a diagonal of the IFC square (Figure 3). Such behavior can be explained by atomic disorder at these positions. Assuming the presence of S_2_ species, the elongation of the ellipsoids may indicate dimers with significant variations in S–S bond length.

Structural data for the samples discussed below are available in the Cambridge Structural Database: CSD 2581398–2581405.

### 3.3. Photoluminescence Spectroscopy (PL)

Natural, thermally untreated samples from Mishevsko (photoluminescent) and the Urdini Lakes (non-luminescent), as well as heated photoluminescent scapolites from both localities, were analyzed. Photoluminescence (PL) emission and excitation spectra for all samples were measured at room temperature with a FluoroLog3-22 (2014) spectrofluorometer (Horiba Jobin-Yvon, Kyoto, Japan; located in Institute of Optical Materials and Technologies-BAS, Sofia, Bulgaria).

### 3.4. Electron Paramagnetic Resonance (EPR)

Samples of natural, untreated scapolites from Mishevsko (photoluminescent) and the Urdini Lakes (non-luminescent) were analyzed. Samples from both localities heated to 1000 °C were also analyzed, with a photoluminescent specimen selected from the Urdini Lakes. The EPR analysis was performed on a Bruker BioSpin EMXplus10/12 instrument (Karlsruhe, Germany, located in Institute of General and Inorganic Chemistry-BAS, Sofia, Bulgaria). All measurements were conducted in the X-band at a frequency of electromagnetic radiation of 9.45 GHz. For low-temperature measurements, the temperature-variable system ER4141VTM was used. The UV irradiation experiment was carried out using a 100 W Hg lamp (LSB610 type).

### 3.5. Vibrational Spectroscopy

Fourier-transform infrared (FTIR) spectra of powdered samples of natural, untreated scapolites from Mishevsko (photoluminescent) and the Urdini Lakes (non-luminescent) were recorded using a Bruker Tensor 37 spectrometer (Germany, located in Institute of Mineralogy and Crystallography-BAS, Sofia, Bulgaria) in the spectral range of 400–4000 cm^−1^, with a spectral resolution of 4 cm^−1^. The spectra were acquired from KBr pellets by averaging 128 scans.

Raman spectra of the same samples were collected from polished sections using a LabRAM HR Raman spectrometer (Horiba Jobin Yvon, Kyoto, Japan, Institute of Mineralogy and Crystallography-BAS, Sofia, Bulgaria) equipped with an Olympus BH2 microscope (Japan). Measurements were performed on spots approximately 2 μm in diameter using a 633 nm He–Ne laser. Spectra were recorded in the backscattering geometry with a 50× objective over the spectral range of 100–1200 cm^−1^. Spectral processing and figure preparation were carried out using the OriginPro 9.0 software (OriginLab Corporation, Northampton, MA, USA).

### 3.6. Thermal Analyses (DSC-TG)—Mass Spectrometry (MS)

The thermal behavior of untreated samples from both localities was investigated using a DSC-TG analyzer SETSYS2400, SETARAM (France, located in Institute of Mineralogy and Crystallography-BAS, Sofia, Bulgaria), under the following conditions: an atmosphere of Ar flow (30 mL min^−1^); a heating rate of 10 ° min^−1^. About 10 mg of each sample was placed in a corundum crucible, and another empty crucible was used as a reference. An empty crucible run for baseline slope correction of both experimental DSC and TG signals at the same set profile was applied. Simultaneous analysis for detection and quantification of the evolved gases was performed via an OmniStarTM PFEIFFER mass spectrometer (France), coupled to the DSC-TG analyzer by a transfer line heated to 180 °C. The intensities related to the mass-to-charge ratio (*m*/*z*) values of the following species, OH^−^ (17), CO_2_ (44), Cl^−^ (34, 35) and SO_2_(64), were examined. Specialized CALISTO thermal analysis software Version 1.494 was used to conduct the experiments and for data processing.

## 4. Results and Discussion

### 4.1. Single-Crystal X-Ray Diffraction (SCXRD) Analyses

Table 1 and Table 2 present the main parameters of the single-crystal diffraction experiments, reliability factors and structural parameters of representative samples of the studied materials. Laboratory-untreated samples from both localities were measured at low (Msh-LT and UL-LT) and room (Msh-RT and UL-RT) temperatures. Heated samples were measured at room temperature. Two samples from Mishevsko—containing (Msh-H1-RT) and not containing (Msh-H2-RT) SO_4_^2−^ groups—and two samples from the Urdini Lakes—exhibiting (UL-H1-RT) and not exhibiting (UL-H2-RT) luminescence—are presented (Table 2). The obtained stoichiometric formulas correspond to the previously reported chemical analysis data [14,15,34] and define the samples from both localities as meionites in the range of Me57–Me71. The chlorine content, varying from 0.08 to 0.24 apfu, was higher in the samples from Mishevsko, whereas for the samples from the Urdini Lakes, the Cl content was about 0.04 apfu. Sulfate groups were present in all studied samples from the Urdini Lakes and in only one of the measured samples from Mishevsko. Sulfur dimers were identified in all the studied samples, with the highest contents noted for samples from Mishevsko. Regarding the charge imbalance, we can comment that in some samples, an excess of positive charge was observed, which may be an indication of the presence of vacancies among the IFCs instead of the full occupancy of these positions assumed in this study. OH^−^ groups, the presence of which can be verified by the results of the thermal–mass spectroscopic and IR analyses, can probably compensate for the observed excess of negative charge. The unit-cell volumes for Mishevsko samples were larger than those of samples from the Urdini Lakes.

The distances and angles characterizing the geometry of the aluminosilicate framework were comparable to those of previously described natural scapolites crystal structures. Table 3 and Table 4 list the geometric parameters of the atomic species populating the SACs. The following notations are used in the studied structures: S—sulfur in the A-position (SO_4_); S1—dimer sulfur; O7—oxygen of the CO_3_ group; and O8 and O9—oxygen of the SO_4_ group.

Carbon–oxygen distances of 1.33–1.34 Å are typical of the carbonate group (Table 3 and Table 4). Slight elongation of this bond was observed in the heated samples. Distances of about 1.5 Å and O8-S-O8 and O9-S-O9 angles close to 109° suggested the presence of SO_4_ groups in the following samples: Msh-H1-RT, UL-LT, and UL-H2-RT. Significant deviations from these values were observed for UL-RT and UL-H1-RT. Similar deviations, especially with regard to the angles in the sulfate tetrahedron, have been found in already published structures of natural scapolites [16]. A possible explanation is the presence of other sulfur species in SACs. The S1-S1 distance varied, and for individual, heated samples, it decreased. This can be explained by an increase in the (S_2_^•−^) radical amount at the expense of S_2_^2–^ ions. Similar observations have been made by [11].

The data from Table 1, Table 2, Table 3 and Table 4 illustrate a relatively wide range of compositions of the studied scapolites, even within a single locality. This is particularly true for the species populating the SACs. For IFAs, the picture is further complicated by the species diversity and their low contents. This makes correct identification and quantification based on X-ray diffraction data a non-routine task, often requiring additional information obtained through other analytical methods. The proposed refinement approach allows for more precise determination of the amount and type of anionic groups, as well as of the possible positions of the S-S dimers.

The items below are those that we believe deserve attention in the structural determination of scapolites:

(i) Upon refinement of the CO_3_ group, we do not apply the model proposed by [21,26] because it does not correspond to our experimental data. This model suggests a displacement of the carbon atom from the central A-position and three equally occupied positions for its oxygen atoms. We do not observe a displacement of the carbon atom from the A-position, nor do we find suitable oxygen positions for the disordered CO_3_ group. Instead, in the ab-plane, we identify two different electron density maxima distanced at approx. 1.35 and 1.1 Å from the center of the SAC, correspondingly. Based on their parameters, the first one is ascribed to the oxygen atoms surrounding a carbon atom located at the A-position, and we further designate them as O7. The other is identified as S1, indicating the possible position of the sulfur dimer within the structure. Therefore, our model does not require constraints to satisfy the equivalence conditions for angles, distances, and site occupancies in the CO_3_ group.

(ii) The presented approach has also been applied to samples of natural scapolites from other Bulgarian localities, as well as from some world-famous deposits of the mineral such as Slyudyanka (Ural, Russia) and Madagascar. For all of them, with the exception of the latter locality, a low residual electron density was found at a distance of about 1.05–1.15 Å from the central A-position, which, in this work, we denote as S1. It can only be surmised that a similar electron density was observed at the indicated location in previous studies as well, but the authors designated it as the position of an oxygen atom from a disordered CO_3_ group.

(iii) The presence of SO_4_^2−^ groups can be reliably established by the manifestation of the appropriate electron density outside the ab-plane. It is supposed to correspond to the characteristic tetrahedral configuration of the oxygen atoms. Due to its low content and doubt of its existence, a good indicator of its presence is the direction of elongation of the thermal ellipsoids of the oxygen atoms from the carbonate group along the c-axis.

### 4.2. Photoluminescence Spectroscopy (PL)

**Figure 4:**
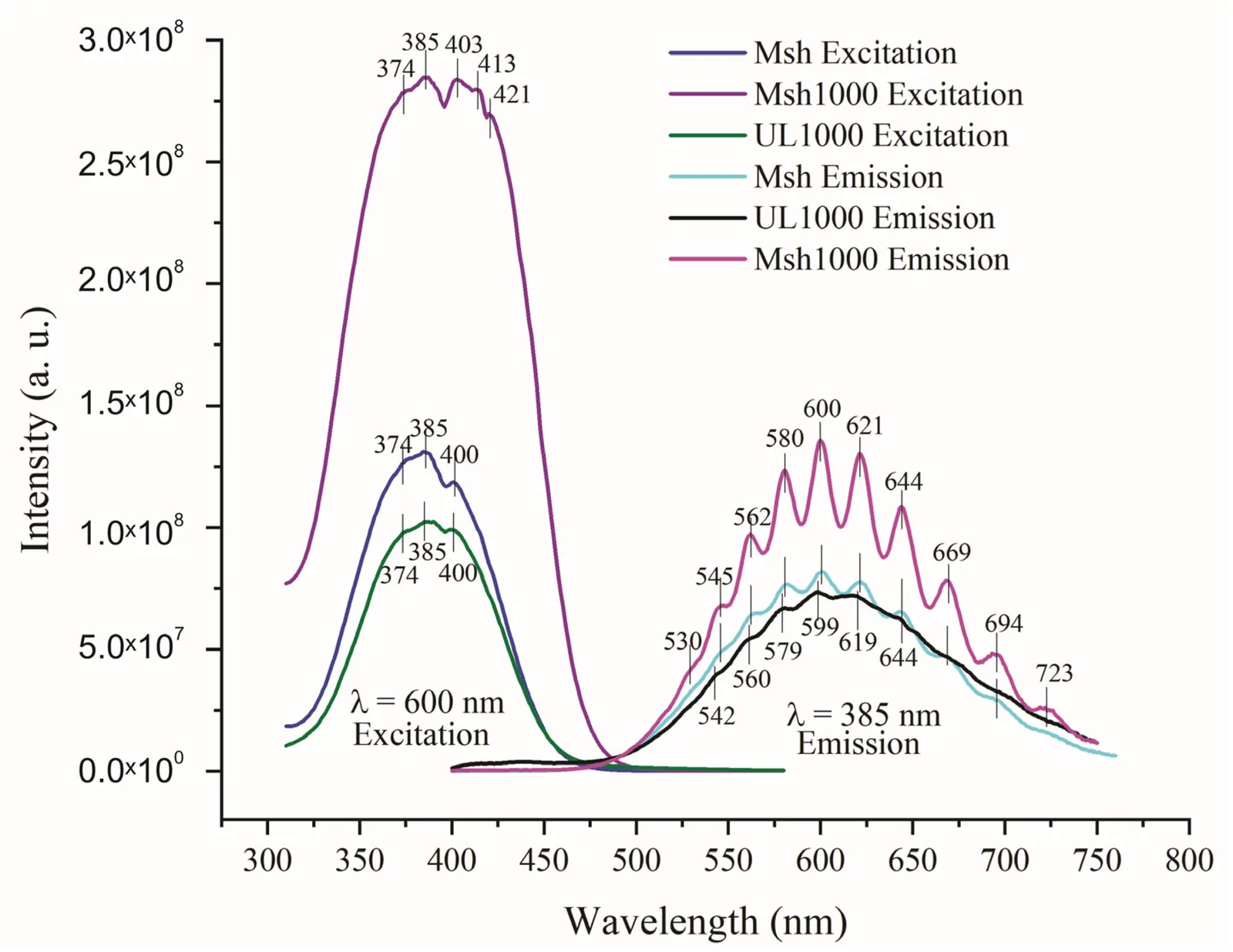
Excitation and photoluminescence emission spectra at room temperature of the studied samples.

The excitation spectra at an emission wavelength of 600 nm and the fluorescent emission at 385 nm excitation at room temperature of the three scapolite samples, natural scapolite from Mishevsko, and thermally treated at 1000 °C scapolites from Mishevsko and the Urdini Lakes, are presented in Figure 4. The excitation spectra of natural (Msh) and heat-treated (UL1000) scapolite samples are very similar, with peaks at 374 nm, 385 nm, and 400 nm, while in the excitation spectrum of the Msh1000 sample, besides these peaks, new peaks at 413 nm and 421 nm can be noticed.

The non-emissive natural UL scapolite after temperature treatment exhibits a fluorescent emission almost identical to that of natural scapolite from Mishevsko. Both present a continuous broad emission spectrum centered at 600 nm in the range between 500 and 740 nm. However, some of the peaks are more clearly defined for the sample from Mishevsko. Figure 4 illustrates that upon heating, the non-emissive natural UL sample began to fluoresce, and for the naturally emitting Msh sample, the intensity of emitted light increased significantly. Distinct peaks at 530 nm, 545, 562, 580, 600, 621, 644, 669, 694, and 723 are characteristic of Msh1000, while those peaks for UL1000 and natural Msh are not as well expressed.

**Figure 5:**
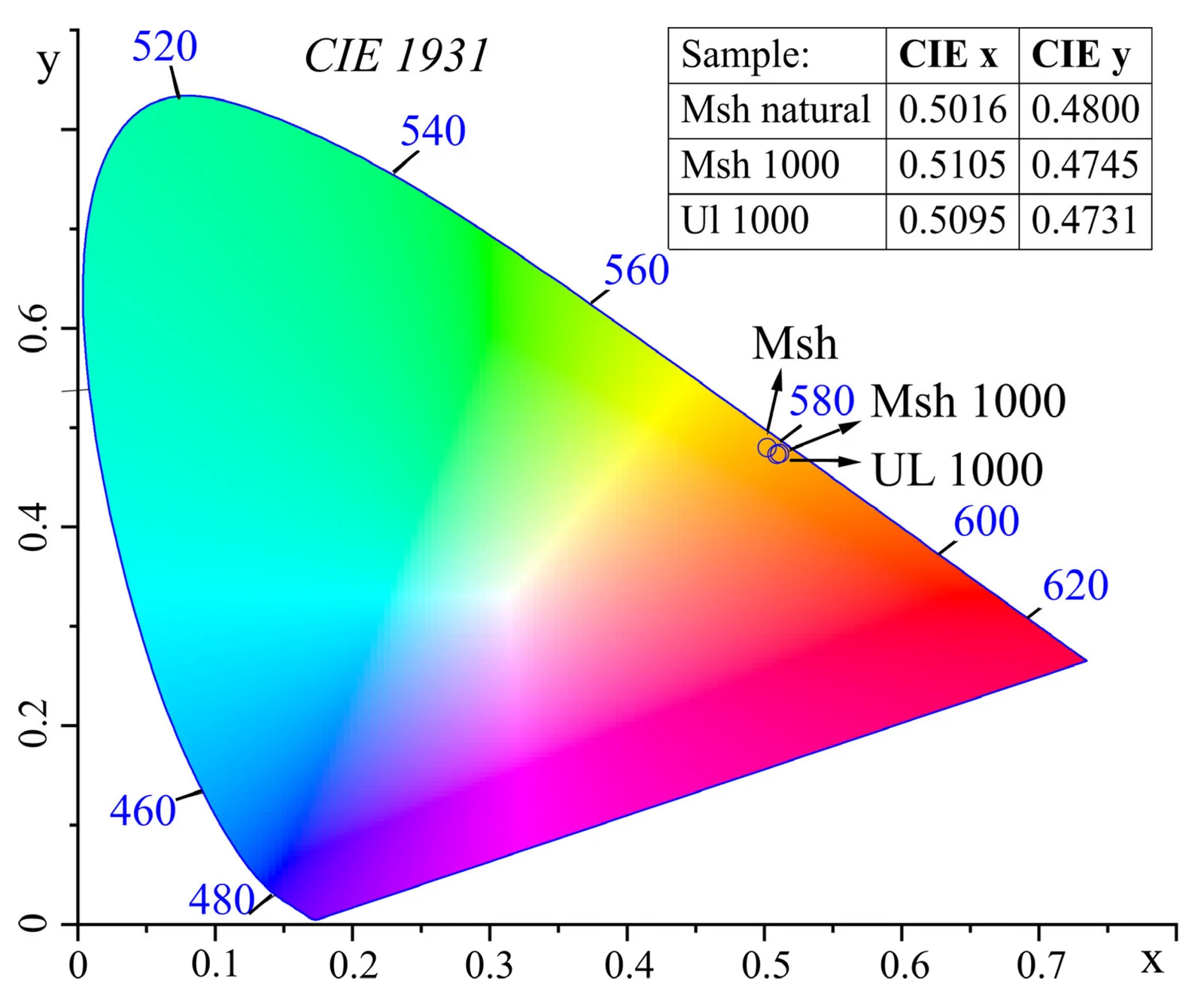
CIE 1931 chromaticity diagram showing the color coordinates of the studied scapolite samples.

The obtained chromaticity coordinates of the samples overlap in the orange part of the color space (Figure 5).

Considering the appearance of photoluminescence at UL1000, as well as the increased emission intensity observed for Msh1000, it can be assumed that the scapolite fluorescent form is more energetically advantageous and stable than the non-fluorescent one. Heating probably alters the concentration and/or electronic configuration of the luminescence centers and enhances excitonic recombination and emission.

The obtained emission spectra closely resemble those reported previously, consisting of a broad band between approximately 475 and 725 nm with a well-resolved vibronic structure [10,12,18,36]. This spectral signature is consistent with electronic transitions associated with S_2_^•−^-related luminescence centers and yellow–orange emission. Based on Steudel’s formula [10,13], the S–S distances for S_2_^•−^ in the studied samples were calculated (Supplementary Materials Table S1 and Table S2). For the Mishevsko sample, these distances ranged from 2.02 to 2.08 Å, while for the Urdini Lakes sample, they ranged from 1.97 to 2.18 Å. The mean value for both samples was 2.04 Å, similar to the one calculated by [10].

### 4.3. Electron Paramagnetic Resonance (EPR)

**Figure 6:**
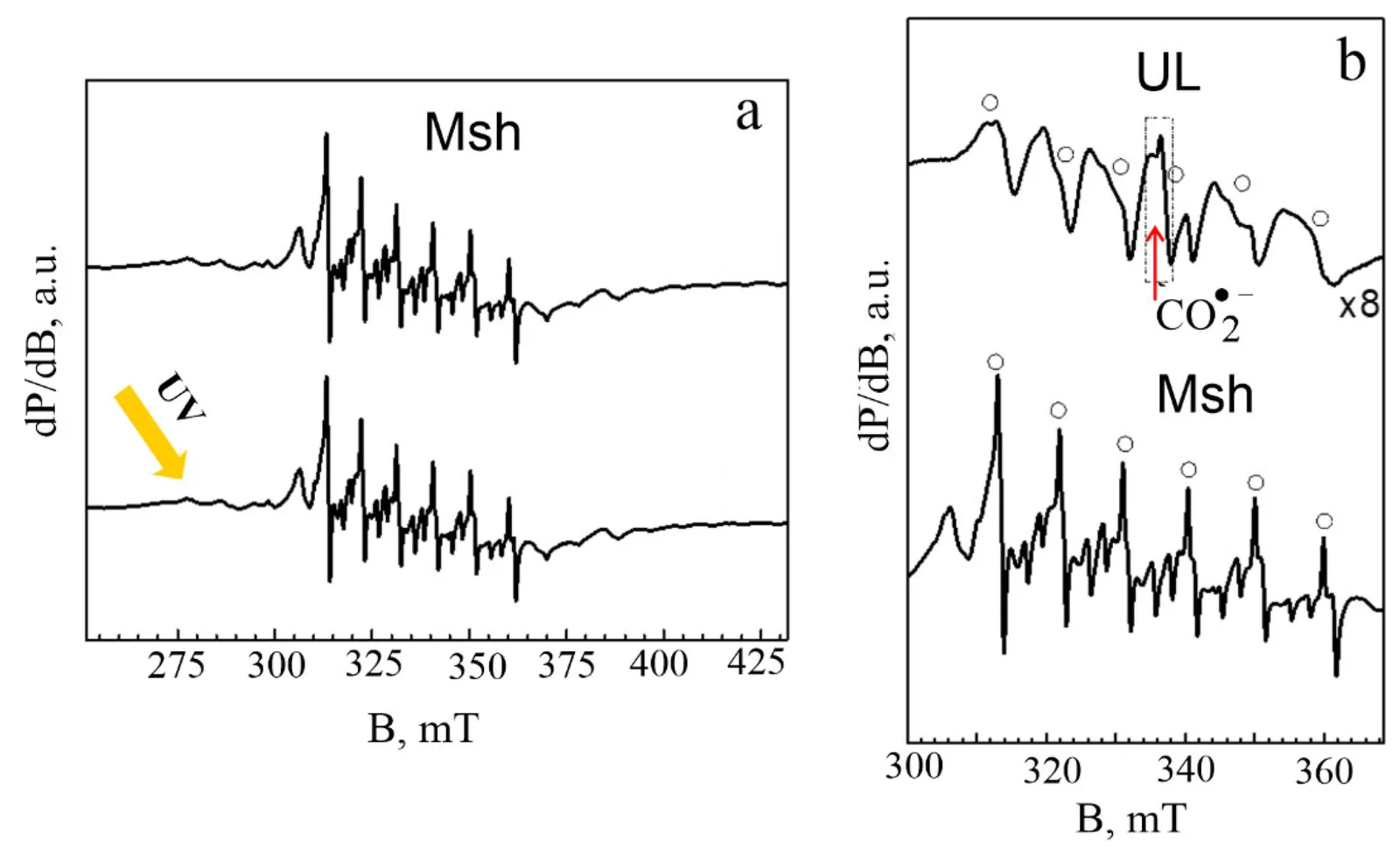
(**a**) EPR spectra of scapolite powder from Mishevsko before and after UV irradiation. (**b**) Comparison of EPR spectra of scapolite samples from the Urdini Lakes and Mishevsko. Open circles indicate the lines due to the Mn^2+^ hyperfine structure. All spectra were acquired at 295 K.

**Figure 7:**
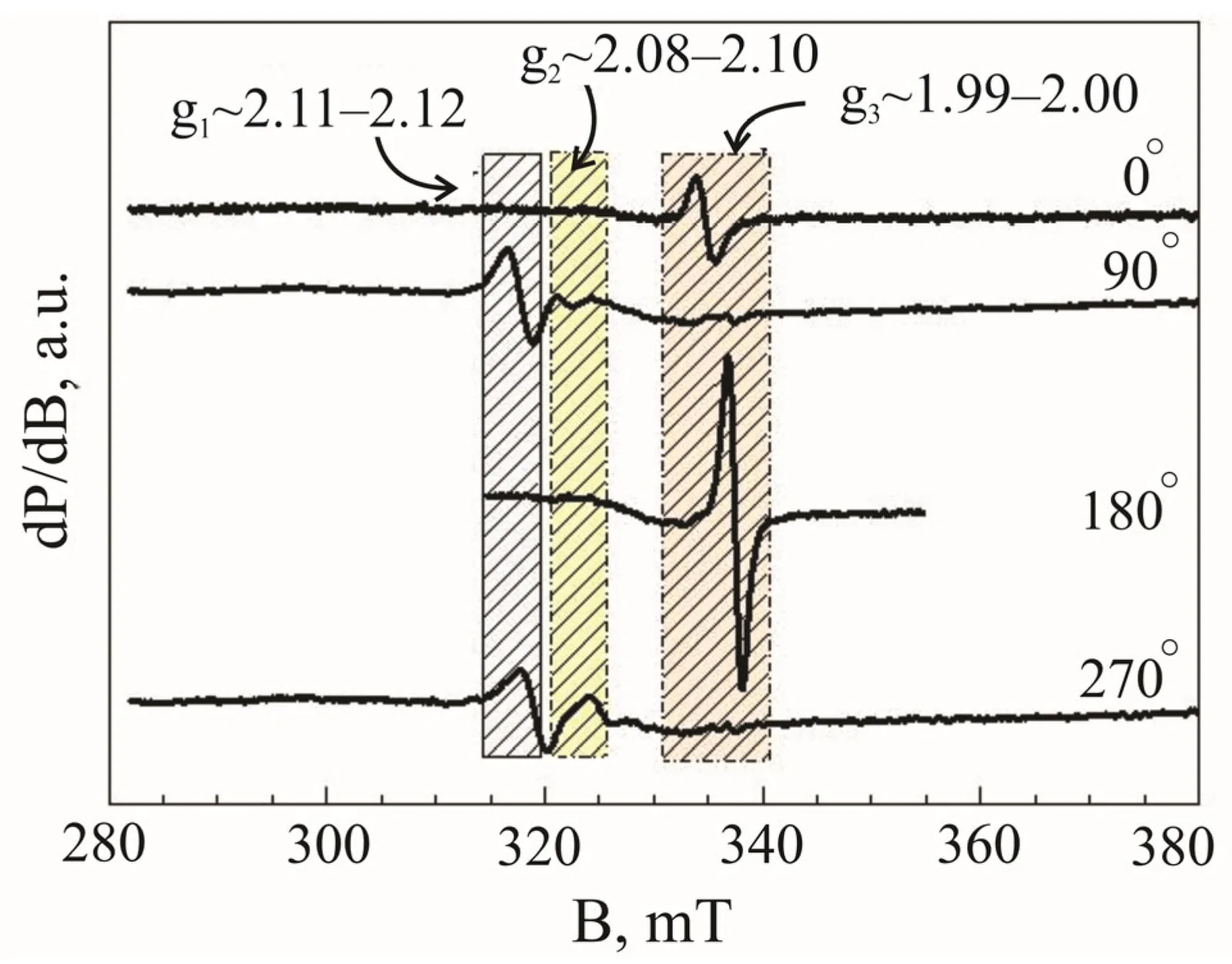
EPR spectra of Msh scapolite fragment. Four EPR spectra are shown that correspond to the orientation of the quartz tube along the magnetic field.

**Figure 8:**
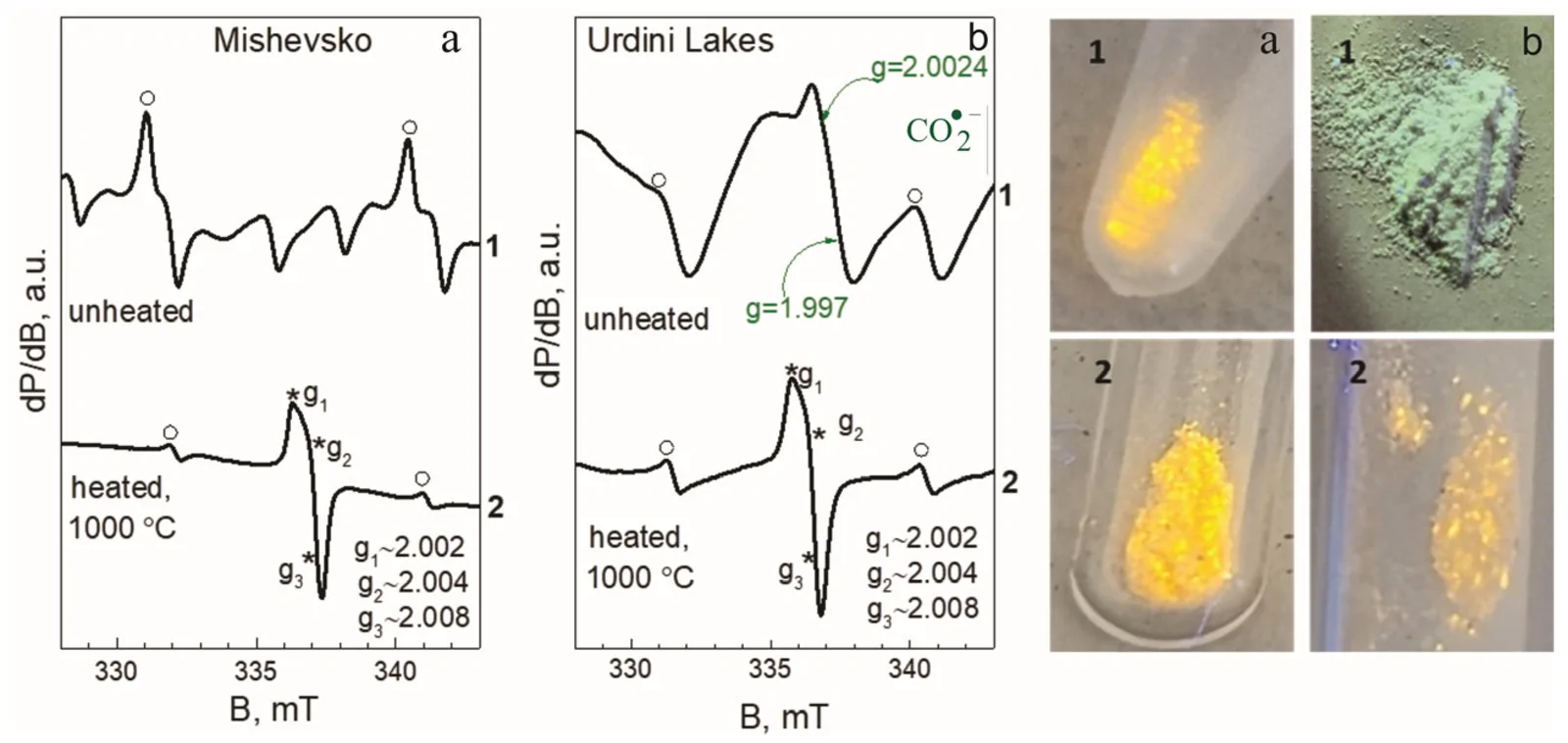
EPR spectra of the studied samples. The 3rd and 4th lines of the Mn^2+^ hyperfine structure are marked by open circles; pictures of UV-irradiated powdered samples corresponding to the EPR spectra are shown on the left. The exact positions of g are denoted by *.

The EPR analyses performed aimed to identify the radical species responsible for the intense yellow luminescence observed under ultraviolet irradiation. Figure 6 and Figure 7 show the spectra of thermally untreated samples, while Figure 8 compares the analytical data for unheated and heated samples from both localities. As seen in Figure 6, the Msh spectrum consists of six main lines accompanied by less intense satellites. These are attributed to isolated Mn^2+^ ions which, as evidenced by chemical analysis [15], are ubiquitously distributed throughout the mineral. The EPR spectrum is unaffected by UV radiation, implying that no photo-induced changes, specifically the generation of radical species, occurred in the studied material (Figure 6a).

As observed in Figure 6b, the UL spectrum also contains the characteristic six-line pattern related to isolated Mn^2+^ ions [34]. The comparison reveals that the lines in the UL sample are 3–4 times broader. This broadening occurs due to less intense forbidden transition lines overlapping with the main hyperfine structure (HFS) lines, indicating that Mn^2+^ ions in the Urdini Lakes occupy a highly distorted environment. To avoid interference from the Mn^2+^ HFS, several Msh crystalline samples were analyzed to select manganese-free materials. Figure 7 shows the Mn-free EPR spectra of about 0.1 cm sized fragments. Four measurements were taken while rotating the quartz cuvette in the plane of the magnetic field. Consequently, two types of EPR signals emerge: at arbitrarily designated 0° and 180° orientations (which are equivalent with respect to the magnetic field vector), a signal with a g-factor of approximately 1.99–2.00 is observed. At the two adjacent orientations (90° and 270°), two distinct signals are registered, with g-factors ranging from 2.11 to 2.12 and 2.08 to 2.10 (g_av_ ~ 2.074) (Figure 7).

Due to the polycrystalline nature of the sample and an approximately defined rotation angle with respect to the magnetic field, the g-factor falls within a range of values and cannot be fixed at a single absolute value.

It should be pointed out that the obtained spectra at each orientation resemble a single-crystal spectrum rather than a polycrystalline one. Such behavior arises from the non-random orientation of microcrystals within the bulk segment. The superposition of all signals registered in a single fragment would form the anisotropic spectrum of a paramagnetic center. The experimentally found g-factor values are in good agreement with earlier presented values for the rhombic symmetry of S^•−^ in scapolite, namely, g_1_ = 2.165, g_2_ = 1.996 (g_av_ = 2.108) [37], and alkali halide samples (g_av_ = 2.136) [38] (Supplementary Materials Table S3). The EPR study of unheated scapolite from Mishevsko revealed the existence of the monosulfide radical anion S^•−^, which, so far, has not been reported to cause luminescence.

The EPR spectrum of the unheated sample from the Urdini Lakes (Figure 8) possesses a signal due to the CO_2_^•−^ anion with axial symmetry, where g_II_ = 1.9970 and g_⊥_ = 2.0024 (g_av_ = 2.0006). These values correspond closely to those reported earlier for rhyolite after gamma irradiation: g_II_ =1.9975, g_⊥_ = 2.0028, and g_av_ = 2.0010 [39]. For comparison, the value of g_av_, determined for CO_2_^•−^ in SrC_2_O_4_ [40], is 2.0019. It should be mentioned that a characteristic feature of the carbon dioxide anion radical is an average g-factor value lower than the free electron value: g_e_ = 2.0023.

Heating at 1000 °C in air induced changes in the scapolite from Mishevsko, leading to suppression of the intensity of Mn^2+^ lines and the disappearance of the anisotropic signal related to the monosulfur radical anion, S^•−^ (g_av_ ~ 2.074). Furthermore, a new EPR signal appears. It features an anisotropic profile with the following g-factors: g_1_ = 2.002, g_2_ = 2.004, and g_3_ = 2.008. The reported signal seems to be related to the heating process because it appears only in the samples thermally treated at 1000 °C. Although it is tempting to identify the heat-induced EPR signal as the source of luminescence, the unheated Mishevsko scapolite exhibits intense luminescence even though its spectrum is free of the suspected signal. Therefore, this signal bears no relation to the observed luminescence properties. Taking these considerations into account, we find it more reasonable to relate this signal to sulfur-centered radical anions, specifically SO_2_^•−^ [41,42].

Thus, we conclude that the luminescence and the discussed heat-induced signal are unrelated phenomena. It should be noted that EPR studies on S_2_^•−^ radicals have been reported for various aluminosilicates, including members of the zeolite, sodalite, and cancrinite groups [10,18,30,43,44,45]. However, the g-factor values reported for the S_2_^•−^ radical in scapolite are based solely on theoretical calculations [12] (Supplementary Materials Table S3). In 2022, a signal with EPR parameters (g_1_ = 2.008 and g_2_ = 2.001) was related to a disulfide anion radical in sapozhnikovite, a mineral from the sodalite group [46]. EPR studies on yellow chromophore S_2_^•−^ in ultramarine pigment revealed remarkably high experimentally found g-factor anisotropy, with g_1_ = 2.69(6), g_2_ = 2.03(4), and g_3_ = 1.86(4) (g_iso_ = 2.19) [47], while computational models predicted slightly higher values of g_1_ = 2.858, g_2_ = 2.034, and g_3_ = 2.002 (g_iso_ = 2.298) [43]. In zeolite and sodalite frameworks, S_2_^•−^ is associated with two-component spectra consisting of broad and narrow lines, respectively, with g-factors of 2.4 and 2.00 [44]. Notably, detection of this disulfide radical anion is typically restricted to temperatures ≤ 30 K due to its large anisotropy and degenerate π-orbitals. In this context, it was assumed that the room-temperature detection of S_2_^•−^ in cancrinite was due to the smaller volume of ε-cages, which retarded their mobility [48]. The authors assigned an EPR signal with an isotropic g-factor close to 2.009 at room temperature and 2.0005 at 77 K to the disulfide anion radical.

Based on the literature data, the luminescent samples showed no S_2_^•−^ radical anion signal, most likely because measurement at temperatures below 30 K is required.

### 4.4. Vibrational Spectroscopy (Infrared and Raman)

#### 4.4.1. Infrared Spectroscopy

**Figure 9:**
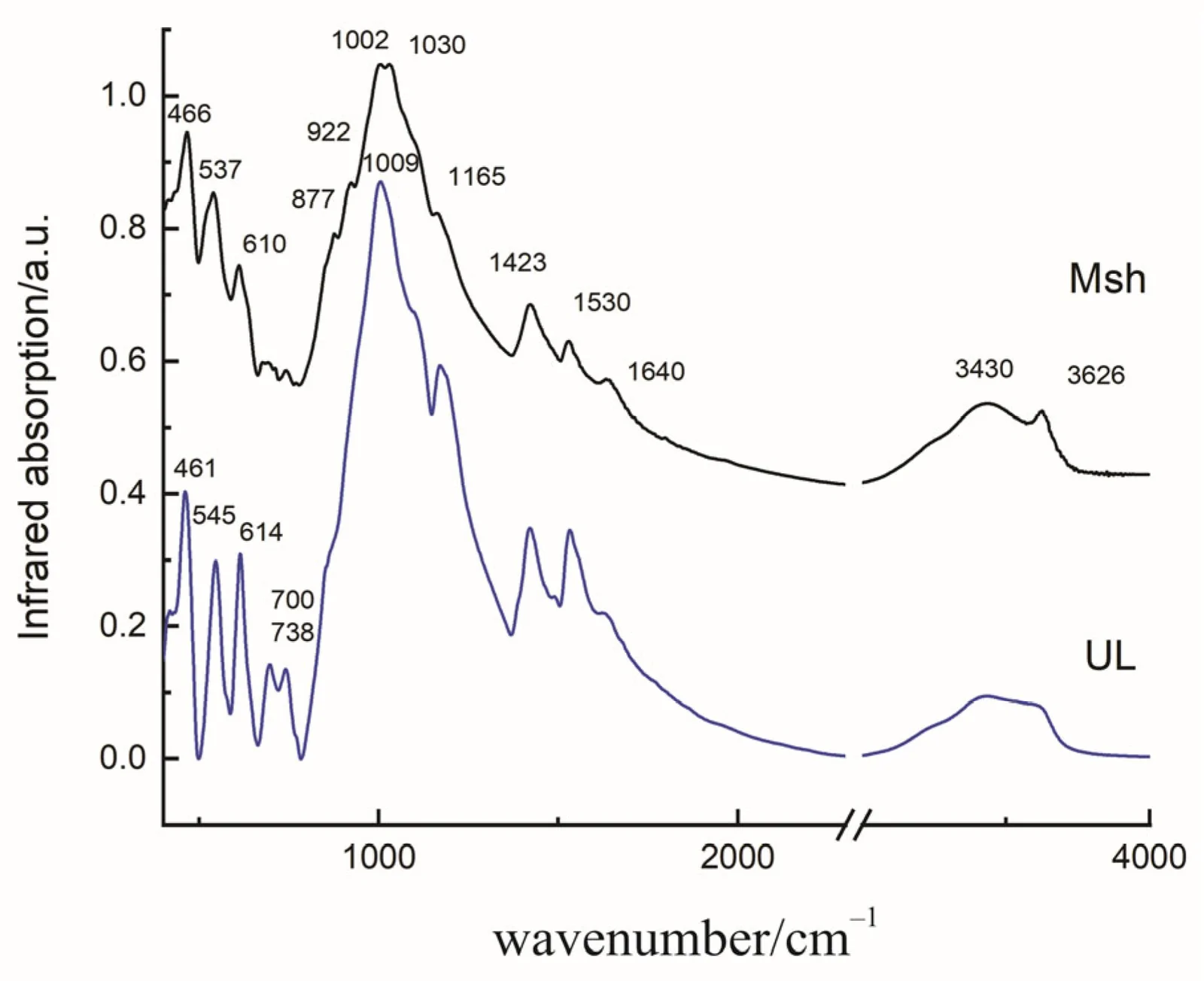
FT infrared spectra of the studied samples.

**Figure 10:**
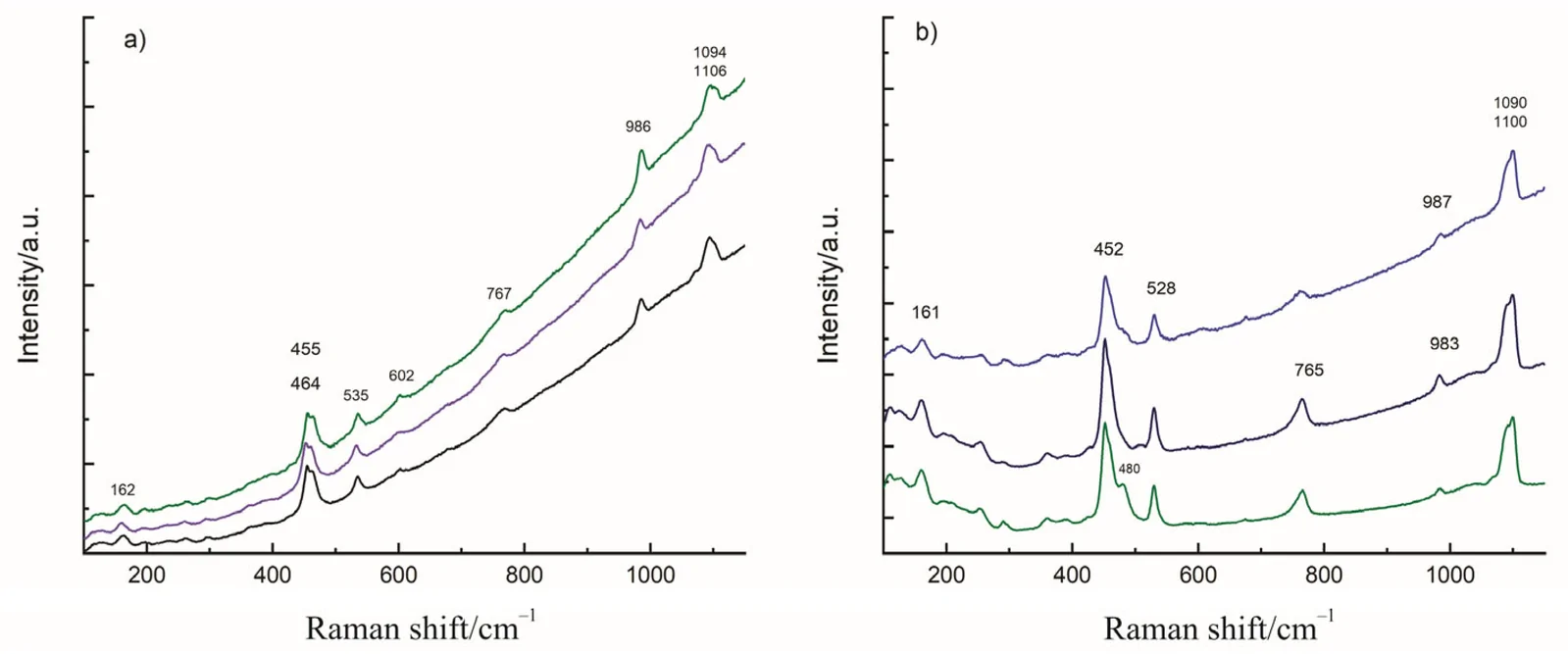
Raman spectra of the studied samples. (**a**) Msh; (**b**) UL.

Figure 9 and Figure 10 present the IR and Raman spectra of untreated samples from the Mishevsko and the Urdini Lakes localities. The infrared spectra reveal broad bands at ~1640 and ~3430 cm^−1^, indicating the presence of water molecules, whereas the sharp peak at 3626 cm^−1^ is characteristic of O-H stretching vibrations in structurally bound hydroxyl groups (Figure 9). The bands in the 1420–1530 cm^−1^ range correspond to the anti-symmetric stretching vibrations of the carbonate group, while the band at 877 cm^−1^ corresponds to the out-of-plane bending mode of CO_3_^2−^. The absorption bands between 900 and 1200 cm^−1^ and between 530 and 740 cm^−1^ and near 460 cm^−1^ are attributed predominantly to aluminosilicate framework vibrations, including T-O stretching, O-T-O bending, and T-O-T bending modes (T = Si, Al) [49,50]. Strong overlap between the aluminosilicate framework vibrations and the anti-symmetric stretching ν_3_ of SO_4_^2−^ vibrations results in intense absorption bands in the 1030–1160 cm^−1^ range.

#### 4.4.2. Raman Spectroscopy

The Raman spectra of the studied scapolites are similar to those previously reported [9,12,51,52,53] (see also Supplementary Information Table S4). They are dominated by vibrations of the aluminosilicate framework, with additional contribution from extra-framework anionic groups (Figure 10). The two peaks in the 1090–1100 cm^−1^ region are attributed to overlapping contributions from the symmetric stretching of carbonate groups and Si-O stretching vibrations of the aluminosilicate framework, with possible contributions from ν_3_(SO_4_). The peak at 983–987 cm^−1^ is assigned to the ν_1_ symmetric stretching of SO_4_^2−^ tetrahedra. The peak at approximately 765 cm^−1^ corresponds to tetrahedral breathing vibrations of the aluminosilicate framework. The bands in the 500–620 cm^−1^ region arise predominantly from T-O-T bending vibrations (Si-O-Si and Si-O-Al) within the tectosilicate structure, with possible contributions from SO_4_ bending modes. The bands at 452 and 420 cm^−1^ are assigned to framework O-T-O deformation modes, while the low-frequency region (256–150 cm^−1^) is attributed to lattice vibrations involving Ca, Na and framework translations.

The Raman and infrared data indicate evidence for the presence of CO_3_^2−^, SO_4_^2−^, H_2_O, and OH^−^ among the extra-framework and volatile components of the studied scapolites. No distinct bands that can be unambiguously attributed to sulfur dimer species were identified. Their absence may be related to the low abundance and to the overlap of their vibrational modes with intense bands arising from the aluminosilicate framework and sulfate groups. This interpretation is consistent with the SCXRD data presented in Table 3 and Table 4, which indicate a low concentration of S_2_ species and S–S bond lengths ranging from 1.97 to 2.3 Å. Applying the empirical relationship proposed by [13], the expected stretching frequencies of sulfur dimers in the studied samples are estimated to fall within a broad range of approximately 290 to 630 cm^−1^, which further complicates their identification.

### 4.5. Thermal Behavior

Previous studies on the thermal behavior of scapolites revealed a wide range of volatile component release, without clearly defined trends. The thermal behavior of scapolites across the marialite–meionite series was investigated using TG, DTA, and mass spectrometry (25–1400 °C; high vacuum) [54]. The studied materials showed continuous volatile release, with five main stages corresponding to H_2_O, SO_2_, CO_2_, NaCl, and KCl. In another study, Me79.6 and Me33.3 samples exhibited different thermal behaviors: Me79.6 released CO_2_, minor NaCl, and SO_3_ in two steps before melting at 1407 °C, whereas Me33.3 lost NaCl, CO_2_, and minor SO_3_ in a single step and melted at 1319 °C. Neither H_2_O nor OH groups were confirmed in the latter study [55].

**Figure 11:**
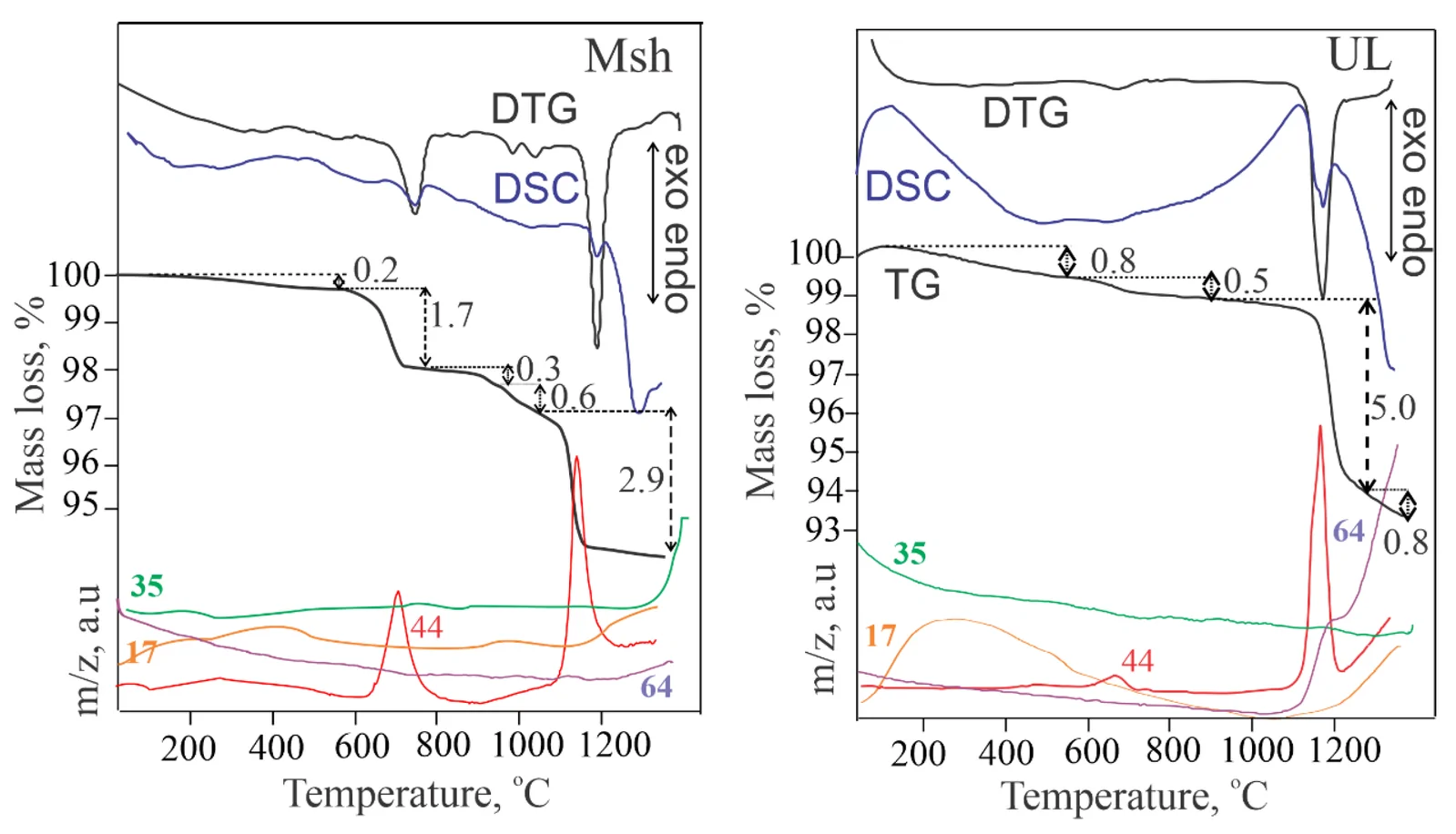
DSC-TG(DTG)-MS data of the studied samples; *m*/*z* = 17 (H_2_O), *m*/*z* = 44 (CO_2_), *m*/*z* = 35 (Cl^−^) and *m*/*z* = 64 (SO_2_).

The thermal behavior of both localities’ samples studied here is expressed by endothermal events on the DSC curves, mass loss recorded on the TG curves, and the corresponding release of volatile components detected by MS (Figure 11). The discussion is based on the stoichiometric compositions obtained after solving the crystal structures of the samples taken at room temperature (Table 1).

UL − ***Na*_1.38_*Ca*_2.62_(*Si*_7.25_*Al*_4.75_)*O*_24_(*CO*_3_)_0.7_(*SO*_4_)_0.16_*Cl*_0.04_(*S*_2_)_0.12_**

A diffuse endothermic effect is observed in the temperature range of 25–500 °C, accompanied by a mass loss of 0.8%. Within this temperature range, the MS curve detects the release of OH ions. The next endothermic event occurs in the temperature range of 600–800 °C, with a negligible amount of 0.5 mass % CO_2_ liberation. Within the range of the last endothermic effect—1100–1300 °C—the release of CO_2_ from the carbonate group is detected, as well as the beginning of the process of release of SO_2_ from the sulfate group. Although the mass loss corresponding to this effect is due to both volatile components evolving, we can assume that the release of CO_2_ (5.0 mass %) has a dominant role, and this value correlates well with the stoichiometric presence of CO_3_ per formula unit, as evidenced by the SCXRD data (Table 1 and Table 2). The release of the SO_2_ component continues up to 1350 °C, at which temperature the melting process of the residue occurs. This process is accompanied by the beginning of hydroxyl ion release, probably as a concomitant post-volatile component during melting.

Msh − ***Na*_1.40_*Ca*_2.60_(*Si*_7.53_*Al*_4.47_)*O*_24_(*CO*_3_)_0.65_*Cl*_0.15_(*S*_2_)_0.22_**

The scapolite sample from Mishevsko showed some differences in terms of thermal behavior compared with the sample from the Urdini Lakes. The mass loss associated with the dehydroxylation process in the temperature range of 25–500 °C was significantly smaller, while the mass loss associated with the decarbonatization process in the temperature range of 600–800 °C was higher. However, in this sample, the presence of a negligible amount of calcium carbonate was assumed [15], which also undergoes decarbonization within the framework of this effect. In the temperature range of 900–1350 °C, a three-step separation of CO_2_ from the carbonate group was registered, and it was most significant within 1100–1300 °C. The total mass loss associated with the release of CO_2_ from CO_3_ (3.8 mass %) correlated with the stoichiometric presence of CO_3_ per formula unit (Table 1 and Table 2). This sample was not characterized by the release of SO_2_, but at the end of the experiment, after 1330 °C, chlorine release was initiated from the framework of the melting residue. In this case, OH ion release was also observed during the melting of the sample.

For both samples, the dehydroxylation in the temperature range of 25–500 °C (more pronounced in the UL sample) was due to the presence of OH groups. The release of CO_2_ at lower temperatures, 600–800 °C, was associated with the presence of carbonate groups belonging to calcite. The released amount was higher for the Msh sample. The main process of volatile release occurred in the temperature range of 1000–1350 °C. CO_2_, SO_2_ or Cl^−^ were released, which confirmed the presence of carbon, sulfur and chlorine in the atomic configuration of the specific species populating the SACs.

### 4.6. Considerations Regarding the Occurrence of Thermally Induced Luminescence

The applied combination of complementary analytical methods provides a more comprehensive view of the diversity of extra-framework species in the scapolite anion cages and their possible transformations during thermal treatment. This is particularly evident for scapolites from the two Bulgarian localities: the Urdini Lakes and Mishevsko. Some of the methods (SCXRD and EPR) were also used to study non-fluorescent scapolites from other Bulgarian sites, such as Samurski Dol and Vacha Reservoir in the Rhodope Mountains, as well as from other locations: Slyudyanka (Urals, Russia); Madagascar; and Obernzell (Bavaria, Germany). All of the samples were heated to 1000 °C in air (with a laboratory furnace) and/or Ar (using thermal analyzer equipment). Regardless of the treatment atmosphere, the heat-treated scapolites exhibited the characteristic orange–yellow photoluminescence under LW-UV excitation (Table S5 + video files in Supplementary Materials). Notably, this phenomenon affected varying volumes of the total material used in the experiment. The single-crystal samples from Slyudyanka and Madagascar exhibited the smallest fluorescent volumes. Scapolites that exhibited characteristics typical of rock-forming minerals, such as polycrystallinity, fractures, and intergrowths with other minerals (e.g., plagioclases and pyroxenes, as seen in the Mishevsko and Urdini Lakes samples), were affected to a greater degree. SCXRD analyses conducted using the described methodology consistently revealed, albeit with varying intensities, electron density in the ab-plane at a distance of approximately 1.1 Å from the A-position in all samples except the unheated Madagascar sample.

The results of the temperature experiments carried out do not confirm the conclusions drawn by Sidike regarding transformations attributed to the alteration of SO_3_^2−^ and SO_4_^2−^ to S_2_^•−^ [18]. There are, however, indications of certain changes in the sulfur species following heat treatment: shortening of the S–S distance (SCXRD); conversion of the S_2_^•−^ radical into other species (EPR and TG-MS); and the formation of new radicals such as SO_2_^•−^ (EPR). Nevertheless, based on the obtained experimental data, some general considerations regarding the formation of S_2_^•−^ radicals in photoluminescent scapolites can be drawn. The presence of S–S dimer precursors in the cages is a necessary condition; however, thermal treatment alone is not sufficient for their transformation into S–S radicals, even though temperature proves to be a key factor. Since only certain parts of the samples fluoresce after heating, an additional condition must be required for the phenomenon to occur. We believe this condition is the predominant presence of sodium near the S-S dimer. This assumption is suggested, albeit indirectly, by the fact that the majority of published data on the luminescence and tenebrescence mechanisms of natural and synthetic scapolites and related aluminosilicates pertain to either pure sodium species or species in which sodium predominates over calcium [4,5,7,10,18]. Sodium atoms in the vicinity of S-S dimer species in SACs are more effective than other cations, e.g., calcium, at capturing and retaining electrons because, despite having similar atomic radii, they possess a lower charge and are, consequently, more mobile within the space defined by the aluminosilicate framework [5]. The formation conditions of the rocks from the above-mentioned Bulgarian localities also suggest that the presence and concentration of sodium are important for the formation and stabilization of S-S radicals that act as photoluminescent centers. The Mishevsko samples originated from rocks that underwent intense fracturing due to tectonic impact after scapolite formation and subsequent albitization [15]. These processes facilitated redistribution of sodium influx, initially taken up by the peripheral zones of the heavily cracked scapolite grains. In contrast, the UL samples were relatively more massive and mechanically intact; the thermally induced photoluminescence was localized mainly in heterogeneities such as fractures, boundaries with other minerals, or other linear or planar defects. A similar phenomenon was observed in the other non-fluorescent scapolites examined. However, targeted studies are needed to establish a systematic relationship between the genetic characteristics of the host rocks and the occurrence of photoluminescence based on obtained emission spectra.

## 5. Conclusions

A new interpretation of the electron density distribution within the scapolite anionic cage is proposed. This interpretation allows for the localization of sulfur monomers or dimers as precursors to the formation of S_2_^•−^ radicals, which emit light under favorable conditions. Parameters such as structural orientation, concentration, and possible bond lengths are determined.

A methodology is proposed to determine the quantities of SO_4_^2−^ and CO_3_^2−^ in SACs based on the occupancy of their oxygen sites. These sites are selected from the electron density distribution map relative to the appropriate geometric parameters (bonds and angles).

The applied combination of complementary structural, spectroscopic and thermal methods provides a more comprehensive understanding of species diversity within the scapolite anion cage, as well as potential species transformations resulting from environmental changes. SCXRD allows for the identification of sulfur species at the level of geometric configuration (dimer). PL analysis can detect only those species that are radicals (S_2_^•−^), regardless of their origin. The EPR method is sensitive to the detection of various sulfur species (e.g., SO_2_^•−^); however, specific analytical conditions are required for the S_2_^•−^ radical.

Conditions that may favor the formation of S_2_^•−^ radicals in fluorescent scapolites are discussed. The results suggest that the presence of appropriate sulfur species together with a suitable local cationic environment and conditions favorable for thermally activated charge redistribution may be required for the development of persistent luminescence.

## Figures and Tables

**Table 1:** Most important data collection and refinement parameters for the unheated samples.

Sample	Msh-LT	Msh-RT	UL-LT	UL-RT
Chemical formula	Na_1.35_Ca_2.65_ (Si_7.44_Al_4.56_)O_24_ (CO_3_)_0.65_Cl_0.24_ (S_2_)_0.15_	Na_1.40_Ca_2.60_ (Si_7.53_Al_4.47_)O_24_ (CO_3_)_0.65_Cl_0.15_ (S_2_)_0.22_	Na_1.38_Ca_2.62_ (Si_7.52_Al_4.48_)O_24_ (CO_3_)_0.73_(SO_4_)_0.13_Cl_0.04_ (S_2_)_0.13_	Na_1.38_Ca_2.62_ (Si_7.25_Al_4.75_)O_24_ (CO_3_)_0.7_(SO_4_)_0.16_ Cl_0.04_ (S_2_)_0.12_
Charge imbalance *	+0.25	−0.16	+0.12	−1.13
Formula weight	910.20	911.45	919.53	919.59
Space group	*P*4_2_/*n*	*P*4_2_/*n*	*P*4_2_/*n*	*P*4_2_/*n*
a = b (Å)	12.1270(16)	12.1474(9)	12.1234(8)	12.1350(7)
c (Å)	7.5688(12)	7.5622(10)	7.5577(6)	7.5560(5)
Volume (Å^3^)/*Z*	1113.1(3)/2	1115.9(2)/2	1110.81(17)	1112.68(15)
ρ_calc_ (g/cm^3^)	2.716	2.713	2.749	2.743
μ (mm^−1^)	1.456	1.1447	1.442	1.436
*Θ* range for data collection (°)	4.75 to 52.928	4.74 to 52.838	4.752 to 52.75	5.496 to 50.484
Data/restraints/parameters	1143/0/111	1144/0/110	1132/0/114	1372/0/114
Goodness-of-fit on *F*^2^	1.080	1.154	1.186	1.038
Final *R* indexes [*I* ≥ 2σ (*I*)]	R_1_ = 0.0405, wR_2_ = 0.1159	R_1_ = 0.0267, wR_2_ = 0.0866	R_1_ = 0.0239, wR_2_ = 0.0792	R_1_ = 0.0438, wR_2_ = 0.0751
Final *R* indexes [all data]	R_1_ = 0.0561, wR_2_ = 0.1298	R_1_ = 0.0349, wR_2_ = 0.0935	R_1_ = 0.0259, wR_2_ = 0.0813	R_1_ = 0.0978, wR_2_ = 0.0925
Largest diff. peak/hole (e^−^ Å^−3^)	0.44/−0.53	0.39/−0.30	0.41/−0.46	0.46/−0.53

* The valence of the sulfur dimer is assumed to be −2.

**Table 2:** Most important data collection and refinement parameters for the heated samples.

Sample	Msh-H1-RT	Msh-H2-RT	UL-H1-RT	UL-H2-RT
Chemical formula	Na_1.18_Ca_2.82_ (Si_7.46_Al_4.54_)O_24_ (CO_3_)_0.66_Cl_0.08_ (SO_4_)_0.12_ (S_2_)_0.18_	Na_1.41_Ca_2.59_ (Si_7.12_Al_4.88_)O_24_ (CO_3_)_0.66_Cl_0.14_ (S_2_)_0.18_	Na_1.71_Ca_2.29_(Si_7.54_Al_4.46_)O_24_ (CO_3_)_0.6_Cl_0.04_(SO_4_)_0.19_ (S_2_)_0.1_	Na_1.38_Ca_2.62_(Si_7.33_Al_4.67_)O_24_ (CO_3_)_0.7_(SO_4_)_0.13_ Cl_0.04_ (S_2_)_0.13_
Charge imbalance *	+0.29	−0.11	+0.01	−0.01
Formula weight	921.12	909.03	907.82	918.64
Space group	*P*4_2_/*n*	*P*4_2_/*n*	*P*4_2_/*n*	*P*4_2_/*n*
a = b (Å)	12.1419(4)	12.1505(3)	12.1153(15)	12.1369(2)
c (Å)	7.5662(4)	7.5632(2)	7.5591(14)	7.5534(2)
Volume (Å^3^)/*Z*	1115.45(9)/2	1116.59(6)/2	1109.5(3)	1112.65(5)/2
ρ_calc_ (g/cm^3^)	2.742	2.654	2.717	2.739
μ (mm^−1^)	1.476	1.355	1.375	1.439
*Θ* range for data collection (°)	6.344 to 57.444	4.742 to 52.856	4.754 to 52.762	4.746 to 52.718
Data/restraints/parameters	1231/0/117	1149/0/110	1133/0/114	1130/0/122
Goodness-of-fit on *F*^2^	1.080	1.095	1.094	1.143
Final *R* indexes [*I* ≥ 2σ (*I*)]	R_1_ = 0.0283, wR_2_ = 0.0912	R_1_ = 0.0279, wR_2_ = 0.0680	R_1_ = 0.0368, wR_2_ = 0.0914	R_1_ = 0.0245, wR_2_ = 0.0771
Final *R* indexes [all data]	R_1_ = 0.0425, wR_2_ = 0.1054	R_1_ = 0.0409, wR_2_ = 0.0794	R_1_ = 0.0496, wR_2_ = 0.1026	R_1_ = 0.0342, wR_2_ = 0.0846
Largest diff. peak/hole (e Å^−3^)	0.34/−0.36	0.44/−0.47	0.38/−0.41	0.29/−0.35

* The valence of the sulfur dimer is assumed to be −2.

**Table 3:** Selected geometrical parameters for the unheated samples.

Sample		Msh-LT	Msh-RT	UL-LT	UL-RT
	Chemical formula	Na_1.35_Ca_2.65_ (Si_7.44_Al_4.56_)O_24_ (CO_3_)_0.65_Cl_0.24_ (S_2_)_0.15_	Na_1.40_Ca_2.60_ (Si_7.53_Al_4.47_)O_24_ (CO_3_)_0.65_Cl_0.15_ (S_2_)_0.22_	Na_1.38_Ca_2.62_ (Si_7.52_Al_4.48_)O_24_ (CO_3_)_0.73_(SO_4_)_0.13_Cl_0.04_ (S_2_)_0.13_	Na_1.38_Ca_2.62_ (Si_7.25_Al_4.75_)O_24_ (CO_3_)_0.7_(SO_4_)_0.16_ Cl_0.04_ (S_2_)_0.12_
distance (Å) and angles (°)					
C–O7		1.339	1.335	1.338	1.356
S1–S1		2.20	2.20	2.228	2.282
S–O8				1.482	1.437
S–O9					
O8–S–O8				108.93	112.97
O9–S–O9					
(Na/Ca)–O7		2.347, 2.447	2.333, 2.470	2.331, 2.443	2.234, 2.538
(Na/Ca)–S1		2.058	2.053	2.050	2.098
(Na/Ca)–O8				2.577, 2.594	2.516, 2.621
(Na/Ca)–O9					

**Table 4:** Selected geometrical parameters for the heated samples.

Sample		Msh-H1-RT	Msh-H2-RT	UL-H1-RT	UL-H2-RT
	Chemical formula	Na_1.18_Ca_2.82_ (Si_7.46_Al_4.54_)O_24_ (CO_3_)_0.66_Cl_0.08_ (SO_4_)_0.12_ (S_2_)_0.18_	Na_1.41_Ca_2.59_ (Si_7.12_Al_4.88_)O_24_ (CO_3_)_0.66_Cl_0.14_ (S_2_)_0.18_	Na_1.71_Ca_2.29_(Si_7.54_Al_4.46_)O_24_ (CO_3_)_0.6_Cl_0.04_(SO_4_)_0.19_Cl_0.04_ (S_2_)_0.1_	Na_1.38_Ca_2.62_(Si_7.33_Al_4.67_)O_24_ (CO_3_)_0.7_(SO_4_)_0.13_ Cl_0.04_ (S_2_)_0.13_
distance (Å) and angles (°)					
C–O7		1.344	1.345	1.344	1.347
S1–S1		2.133	2.098	2.116	2.153
S–O8		1.462		1.264	1.455
S–O9		1.473			1.445
O8–S–O8		106.24		129.01	105.17
O9–S–O9		105.39			104.59
(Na/Ca)–O7		2.333, 2.453	2.342, 2.455	2.310, 2.455	2.233, 2.449
(Na/Ca)–S1		2.082	2.110	2.079	2.066
(Na/Ca)–O8		2.582, 2.646		2.553, 2.493	2.594, 2.639
(Na/Ca)–O9		2.584, 2.652			2.465, 2.784

## Data Availability

The original contributions presented in this study are included in the article/Supplementary Materials. Further inquiries can be directed to the corresponding authors.

## Supplementary Materials

The following supporting information can be downloaded at https://www.mdpi.com/article/10.3390/ma19214623/s1. Table S1: Calculation of the S-S bond length using the vibronic structure of the emission for the Msh sample applying Steudel’s relation for diatomic sulfur species; Table S2: Calculation of the S-S bond length using the vibronic structure of the emission for the UL sample applying Steudel’s relation for diatomic sulfur species; Table S3: EPR parameters of sulfur localized radicals in various minerals, alkali halides, and some chemical compounds [56,57,58]; Table S4: IR and Raman wavenumbers along with their assignments (interpretation) and the sources used for these assignments; Table S5: Heated scapolite samples from various localities under UV light; Figure S1. Rietveld refinement of UL sample heated at 1000 °C (GSAS program); Video clips: Heated_Msh_vs_Unheated_Msh_1.mp4, Heated_Msh_vs_Unheated_Msh_2.mp4, and Heated_UL_vs_Unheated_IL.mp4 are short videos comparing heated and unheated scapolite samples from both localities: Msh and UL.
