# Supplementary material for: Extra-Framework Anion Species in Scapolites Exhibiting Natural and Thermally Induced Photoluminescence: Insights from Single-Crystal XRD
Source: Materials (Basel). 2026 Oct 30;19(21):4623. doi: 10.3390/ma19214623 (PMC13764749; doi:10.3390/ma19214623)
Supplement: Supplementary file 1 [file materials-19-04623-s001.zip › materials-4628547-supplementary.pdf]

Supplementary Information

Extra-Framework Anion Species in Scapolites Exhibiting Natural and Thermally Induced Photoluminescence: Insights from Single-Crystal XRD

Vladislav Kostov-Kytin [1,2,*], Rositsa Kukeva [3], Petia Petrova [4], Rositsa Titorenkova [1,5], Nadia Petrova [1,5], Boris Shivachev [1] and Rositsa Nikolova [1,2,*]

1 Institute of Mineralogy and Crystallography "Acad. Ivan Kostov", Bulgarian Academy of Sciences, Blvd. Acad. G. Bonchev Str., Bld. 107, 1113 Sofia, Bulgaria; rositsatitorenkova@imc.bas.bg (R.T.); nadia5@mail.bg (N.P.); blshivachev@gmail.com (B.S.)
2 National Centre of Excellence "Mechatronics and Clean Technologies", 8 Blvd. Kliment Ohridski, P.C. 1756 Sofia, Bulgaria
3 Institute of General and Inorganic Chemistry, Bulgarian Academy of Sciences, Blvd. Acad. G. Bonchev Str., Bld. 11, 1113 Sofia, Bulgaria; kukeva@svr.igic.bas.bg
4 Institute of Optical Materials and Technologies "Acad. Jordan Malinowski", Bulgarian Academy of Sciences, Blvd. Acad. G. Bonchev Str., Bld. 109, 1113 Sofia, Bulgaria; petia@iomt.bas.bg
5 PERIMED-2, BG16RFPR002-1.014-0007, Central District, Vasil Aprilov Blvd. 15A, 4002 Plovdiv, Bulgaria
* Correspondence: vkytin@abv.bg (V.K.-K.); rosica.pn@clmc.bas.bg (R.N.)

Table S1. Calculation of the S-S bond length using the vibronic structure of the emission for the Msh sample applying Steudel's relation for diatomic sulfur species [13].

Maxima positions (nm)	Maxima positions (eV)	Difference between two successive maxima (meV)	Difference between two successive maxima (cm^{-1})	Calculate **d** bond length (Å)
530	2.339			
545	2.275	64	516.2	2.08
562	2.206	69	556.53	2.04
580	2.138	68	548.46	2.05
600	2.066	72	580.72	2.02
621	1.997	69	556.53	2.04
644	1.925	72	580.72	2.02
669	1.853	72	556.53	2.04
694	1.787	66	532.33	2.07
732	1.715	72	556.53	2.04

Table S2. Calculation of the S-S bond length using the vibronic structure of the emission for the UL sample applying Steudel's relation for diatomic sulfur species [13].

Maxima positions (nm)	Maxima positions (eV)	Difference between two successive maxima (meV)	Difference between two successive maxima (cm^{-1})	Calculate **d** bond length (Å)
nm	eV	meV	cm^{-1}	d(Å)
530	2.339			
542	2.288	51	411.35	2.18
560	2.214	74	596.85	2.00

579	2.141	73	588.79	2.01
599	2.070	71	572.66	2.03
619	2.003	67	540.40	2.06
644	1.925	78	629.12	1.97
669	1.853	72	556.53	2.04
694	1.787	66	532.33	2.07

Table S3. EPR parameters of sulfur localized radicals in various minerals, alkali halides, and some chemical compounds.

	g_1	g_2	g_3	g_{av}	g_{iso}	Matrix	References
$S_2^{\bullet-}$							
4 K	2.69(6)	2.03(4)	1.86(4)	2.190	-	SOD	45 (exp)
	2.858	2.034	2.002	2.298	-	SOD	46 (calc)
298 K	g~2.34 (broad)		g~2.00 (narrow)	-	-	SOD	48(exp)
77 K	2.210	2.010	1.995	2.071	-	SOD	54 (exp)
77 K	2.220	2.007	-	2.078	-	CAN-SOD (Marinelite)	54 (exp)
	-	-	-	-	2.086-2.205	Scapolite with different Na/Ca ratio	10 (calc)
298 K<br><br>77 K	-<br><br>-	-<br><br>-	-<br><br>-	-<br><br>-	2.009<br><br>2.0005	CAN<br><br>CAN	51 (exp)
298 K	2.008	2.001	-	-	-	SOD	49
$S_3^{\bullet-}$							
4 K	2.005	2.041	2.054	2.033	-	SOD	45 (exp)
	2.002<br>2.002<br>2.017	2.038<br>2.039<br>2.02	2.051<br>2.061<br>2.02	2.030<br>2.034<br>2.019	-	SOD<br>*C*2V<br>*D*3*h*	46 (exp)<br>46 (calc)<br>46 (calc)
295 K<br>77 K	2.001	2.035	2.050	2.029	-	LOS	47 (exp)
298 K, 77 K	1.99	2.02	2.2	2.07		CAN	54 (exp)
	-	-	-	-	2.019	CAN-SOD (Marinelite)	54 (exp)
	2.002	2.0378	2.0590	2.0329	-	Scapolite – 4 Na	10 (calc)
	2.0014	2.0308	2.0465	2.0262	-	alkali halide	43 (exp)
293 K, 77 K	2.0338	2.0342	2.0351	2.0345	-	CAN+SOD	51 (exp)
$S^{\bullet-}$							
	2.165	1.996	-	2.108	-	scapolite	39 (exp)
	2.187	2.106	1.996	2.096	-	scapolite	39 (exp)
	2.211	1.986	-	2.136	-	alkali halide	40 (exp)
	2.284	2.543	1.907	2.244	-	alkali halide	40 (exp)
$S_4^{\bullet-}$							
298 K, 77 K	2.020	2.001	-	-	-	SOD	54 (exp) UV-irradiation
	-	-	-	-	2.021	SOD	54 (exp)
$SO_3^{\bullet-}$							
	-	-	-	-	2.004	$Na_2S_2O_6.2H_20$	43 (exp)

	-	-	-	-	2.005	SOD	43 (exp)
	2.002	2.0036	2.0047	2.0034	-	$K_2S_2O_5$	44 (exp)
300 K, 77 K	2.004	2.004	2.004	2.004		$KMgClSO_4.3H_2O$	56 (exp) irradiated
295 K	-	-	-	-	1.9953	$CaSO_4$ anhydrite	57 (exp) γ-irradiated
$S_2O_2^{\bullet -}$							
300 K	2.003	2.005	2.0083	2.0054	-	$Na_2S_2O_3.5H_20$	58 (exp), X-rays irradiated
	2.005	2.005	2.005	2.005	-	SOD	43(exp)
$SSO^{\bullet -}$							
	2.0035	2.0106	2.0287	2.0142	-	$Na_2S_2O_8$.5H20	43(exp)
	2.001	2.011	2.029	2.0136	-		43(exp)
$SO_4^{\bullet -}$							
295 K		$g_{\parallel}$=2.011	$g_{\perp}$=2.012	-	-	$CaSO_4$ anhydrite	57 (exp) γ-irradiated
	2.007	2.011	2.014	-	-	$KMgClSO_4.3H_2O$ kainite	56 (exp) X-rays irradiated
$SO_2^{\bullet -}$							
80-293 K	2.0018-2.0042	2.0048-2.010	2.0056-2.0012	-	-	$Na_2S_2O_5.5H_20$	44 (exp)

Table S4. IR and Raman wavenumbers along with their assignments (interpretation) and the sources used for these assignments

Infrared peak position/ cm^{-1}	Infrared peak position/ cm^{-1}	Mode, assignment	References
3630<br>3430<br>3200		O-H stretching of hydroxyl groups<br>O-H stretching of water molecules/ hydrogen bonded hydroxyl species	[17]
1630 - 1640		H-O-H bending vibrations of molecular water	[17]
1560<br>1530<br>1488<br>1423		Antisymmetric stretching, $\nu_3(CO_3^{2-})$	[9, 12, 17, 49, 50] and references therein
1190 - 1170<br>1002 - 1009		Overlapping contribution of Si-O-Si stretching (SiO_4) and antisymmetric sulphate stretching (SO_4^{2-})	[9, 12, 50] and references therein
	1100 - 1106<br><br>1090 - 1094	Overlapping contribution of Si-O stretching and symmetric carbonate stretching mode ν_1 (CO_3^{2-})	[9, 12, 52, 53] and references therein

	983 - 987	symmetric sulphate group stretching mode, ν_1 (SO_4^{2-})	[9, 12, 52, 53] and references therein
865 - 852		Out of plane bending, ν_2 (CO_3^{2-})	[9, 49, 50]
700		In-plane bending, ν_4 (CO_3^{2-})	[9, 49, 50]
610 - 614		Framework vibration sensitive to composition, T-O-T	[50]
537 - 545<br>521<br>461- 466	528 - 532<br>452 - 455<br>420	Framework deformation vibrations:<br>T-O-T symmetric bending;<br>(SO_4^{2-}) bending;<br>Anion contribution	[9, 52, 53]
	296, 263, 162, 130	Lattice modes involving cations	

Table S5. Heated scapolite samples from various localities under UV light.

<table>
<tr>
<td>Mishevsko.
The non-luminescent parts – calcite
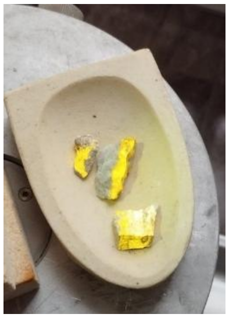</td>
<td>Urdini Lakes.
Massive scapolite -90% and plagioclase – 10 % piece; the same sample – powdered and quantified by Rietveld method (Fig. S1)
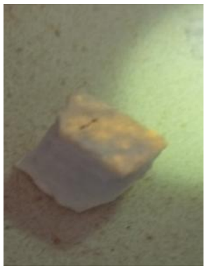 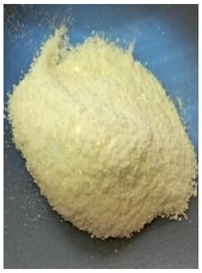</td>
<td>Slyudyanka
Pieces from a specimen taken from the National Museum of Natural History at the Bulgarian Academy of Sciences, Sofia under number 3123 [14].
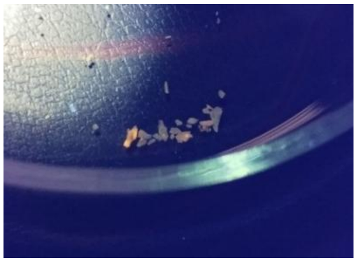</td>
</tr>
<tr>
<td colspan="2">Obernzell (Bavaria, Germany) – left and Madagascar - right
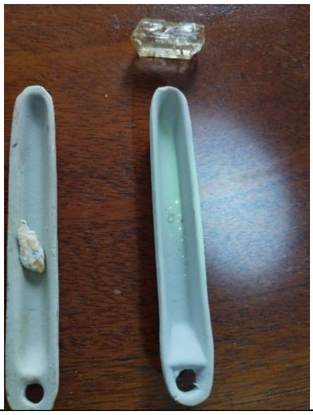</td>
<td>Obernzell (Bavaria, Germany) – upper left; Samurski dol – below and piece of Mishevsko sample.
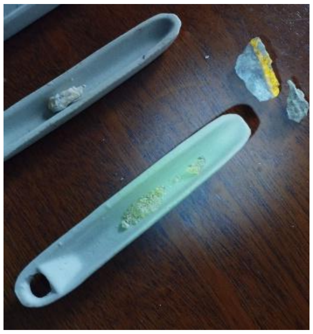</td>
</tr>
</table>

Figure S1. Rietveld refinement of UL sample heated at 1,000 °C (GSAS program).

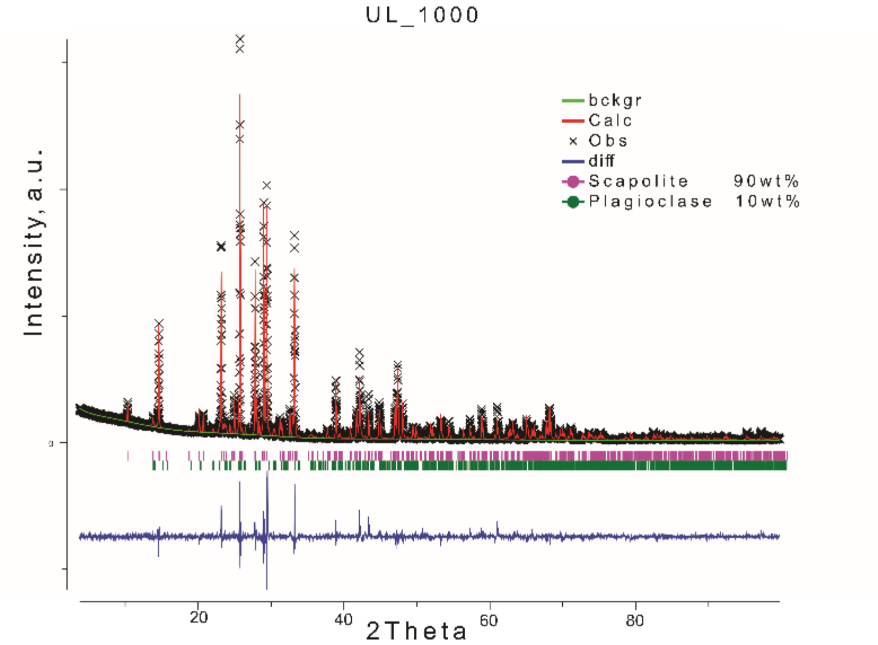

Video clips

Heated_Msh_vs_Unheated_Msh_1.mp4, Heated_Msh_vs_Unheated_Msh_2.mp4, Heated_UL_vs_Unheated_IL.mp4 are short videos comparing heated and unheated scapolite samples from both localities – Msh and UL. In both cases, the heated samples do not fluoresce immediately after being removed from the furnace. The phenomenon begins after 1–2 minutes of cooling and intensifies. A possible explanation – though one requiring more detailed study – is "concentration quenching" and luminescence inhibition, which subside over time due to the conversion of $S_2^{\bullet-}$ into S_2^{2-} or another sulfur species. In our opinion, the fluorescence reaches an intensity greater than that of the thermally untreated scapolite (specifically for the Mishevsko locality). Not all fragments from the Urdini Lakes exhibit fluorescence after heating. For an explanation, see section

4.6. "Considerations regarding the occurrence of luminescence after heating." Where the phenomenon occurs, it remains persistent over time – in the case of the UL samples, for more than five months.
